# State-of-the-Art Review of the Performance of Fiber-Reinforced-Composite-Confined Concrete Columns at Ambient Temperatures

**DOI:** 10.3390/ma18051151

**Published:** 2025-03-04

**Authors:** Zhixin Liu, Chaochao Sun, Jili Qu, Alexander Mokhov

**Affiliations:** 1 School of Environment and Architecture, University of Shanghai for Science and Technology, Shanghai 200093, China; 232291893@st.usst.edu.cn; 2 School of Civil Engineering, Kashi University, Kashi 844000, China; 3 The Southern Scientific Center of the Russia Academy of Sciences, Moscow 4119049, Russia; mochov@ssc-ras.ru

**Keywords:** FRP, stress path, constitutive model, machine learning prediction, compression resistance

## Abstract

This paper investigates the effect of fiber-reinforced composites (FRPs) on the mechanical properties of concrete under ambient conditions. It begins with an examination of the various types of FRP and their advantages, followed by a review of isostructural models for passively restrained concrete under ambient conditions. These models are categorized into two main groups: those assuming constant confining stresses and those that incorporate stress constraints related to the loading history. Recent studies have highlighted the significant role of stress paths in determining the stress–strain behavior of concrete. Traditional methods for predicting the FRP-constrained concrete reinforcement bond at room temperature are increasingly being replaced by machine learning techniques, such as Artificial Neural Networks (ANNs) and Genetic Expression Programming (GEP), which offer superior accuracy in predicting the FRP-constrained concrete bond strength and the compressive properties of FRP-confined concrete columns. In particular, experimental results show that the compressive strength of FRP-confined concrete columns can increase by up to 30–250%. This review offers valuable insights into the effects of FRP on concrete and contributes to the advancement of engineering design practices.

## 1. Introduction

**Figure 1:**
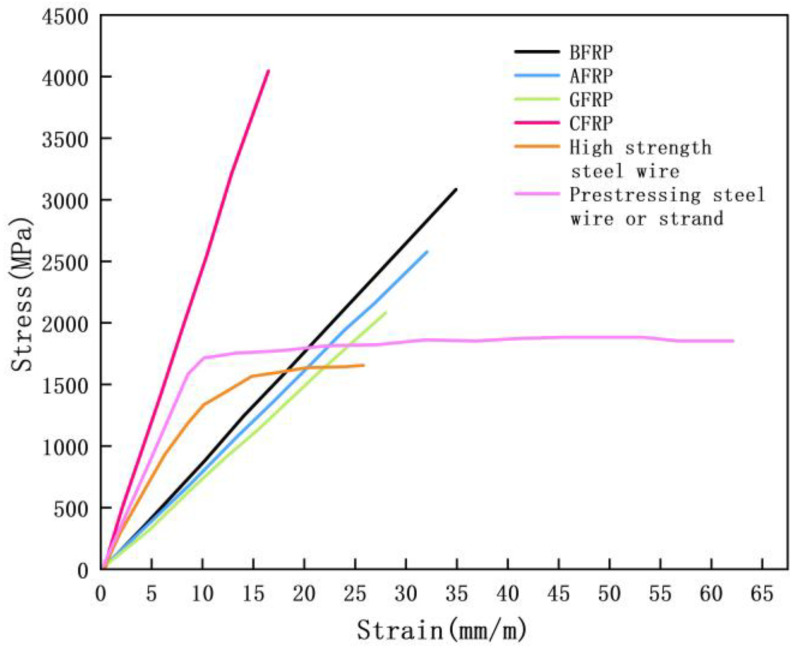
Comparison of FRP material and mild steel [5].

With the continuous developments in advancements in construction materials, fiber-reinforced composites (FRPs) have emerged as a new class of reinforcement materials, widely used in the reinforcement and repair of concrete structures. An FRP is a polymer-based composite material, consisting of continuous fibers or fiber fabrics as the reinforcing phase and polymer resin as the matrix phase, with these two materials combined through a composite process. FRPs can be classified into synthetic fibers and natural fibers based on the fiber type [1,2,3]. Synthetic fibers, such as carbon fiber, glass fiber, and aramid fiber, typically exhibit higher strength and stiffness, making them suitable for applications requiring high strength and durability. In contrast, natural fibers, including hemp, bamboo, and wood fibers, offer better environmental benefits, although they are relatively weaker in terms of strength and durability. The matrix materials used in FRP mainly include epoxy resin and polyester resin [4]. Epoxy resin typically provides superior mechanical properties and chemical resistance, making it ideal for high-performance FRP applications. Polyester resin, on the other hand, is more cost-effective, making it suitable for applications with cost constraints, although it offers lower durability and mechanical performance. Based on the influence of the fiber type on the concrete reinforcement effect, the types of fibers used in FRP include carbon fiber composites (CFRPs), glass fiber composites (GFRPs), aramid fiber composites (AFRPs), and basalt fiber composites (BFRPs). A comparison of FRPs and mild steel is illustrated in Figure 1, and the basic mechanical properties of FRP materials are presented in Table 1.

As can be seen from Table 1, FRP materials exhibit excellent mechanical properties, corrosion resistance, and lightweight characteristics, making them an effective means of enhancing the durability and compressive strength of concrete structures [6,7,8,9]. CFRPs, GFRPs, AFRPs, and BFRPs each have their own advantages and limitations [10,11]. CFRPs offer excellent performance under high strength requirements, making them highly suitable for engineering applications that demand high strength and long-term durability, such as bridges, tunnels, and high-rise buildings. However, the material cost of a CFRP is typically 2–3 times that of a GFRP. The GFRP, while having lower strength and stiffness than the CFRP, offers good toughness and weather resistance, making it a preferred material for many repair projects. The AFRP is generally priced between the CFRP and GFRP, and due to its unique impact resistance and heat resistance, it offers high cost-effectiveness in structures subjected to dynamic loads or extreme environmental conditions. The BFRP, which costs about 70–80% of the CFRP, is an emerging material with good economic and durability properties, particularly suitable for corrosion-resistant and high-temperature applications.

**Figure 2:**
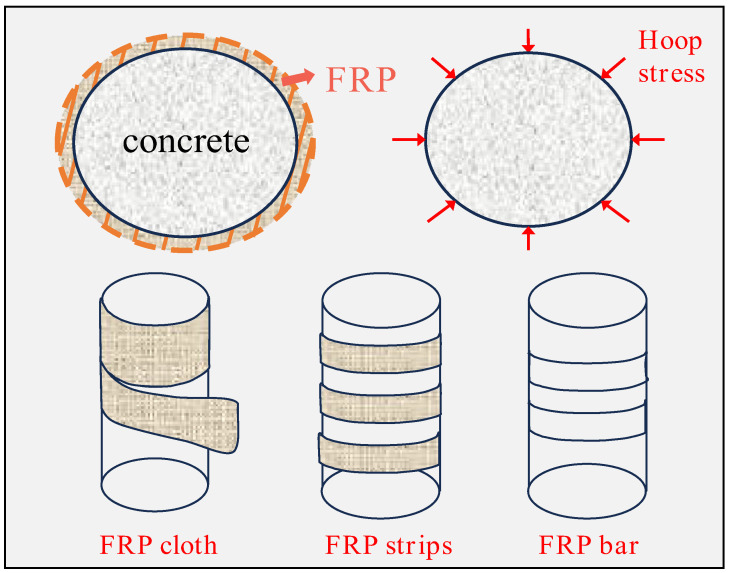
FRP constrains the basic form of concrete columns.

Column structures are one of the most common types of structures in civil engineering, widely used in buildings, bridges, and various other engineering applications. Concrete columns are prone to yielding, failure, or premature damage under axial loads [12,13]. FRP reinforcement can effectively enhance their compressive strength, crack resistance, and shear capacity. In addition, column structures possess relatively simple geometric characteristics in experimental and numerical analyses, making it easier to control variables and conduct precise analyses, thus providing reliable data support for subsequent engineering applications. The basic form of FRP-confined concrete columns is shown in Figure 2.

Current research on FRP-reinforced concrete primarily focuses on the mechanical behavior at room temperature and the prediction of bond performance among FRP, concrete, and steel reinforcement. Although the traditional theoretical models reveal the influence of FRPs on the mechanical properties of concrete to a certain extent, with the deepening of the research, these models increasingly show limitations as research progresses, particularly when considering complex factors, such as the loading history and stress paths. In recent years, artificial intelligence techniques, especially artificial neural networks (ANNs) and gene expression programming (GEP) have introduced new methods for predicting the mechanical properties of FRP-reinforced concrete.

Furthermore, while FRP materials demonstrate excellent performance at room temperature, temperature fluctuations in high-temperature environments significantly affect the performance of FRP-reinforced concrete. The strength and bond properties of FRP materials tend to decrease substantially [14,15,16], which, in turn, impacts the compressive capacity of concrete. Therefore, investigating the mechanical behavior of FRP and concrete under both room and high temperatures, especially the bonding performance and confinement effect between them, will enhance the resistance and fire safety of structures [16,17,18].

**Figure 3:**
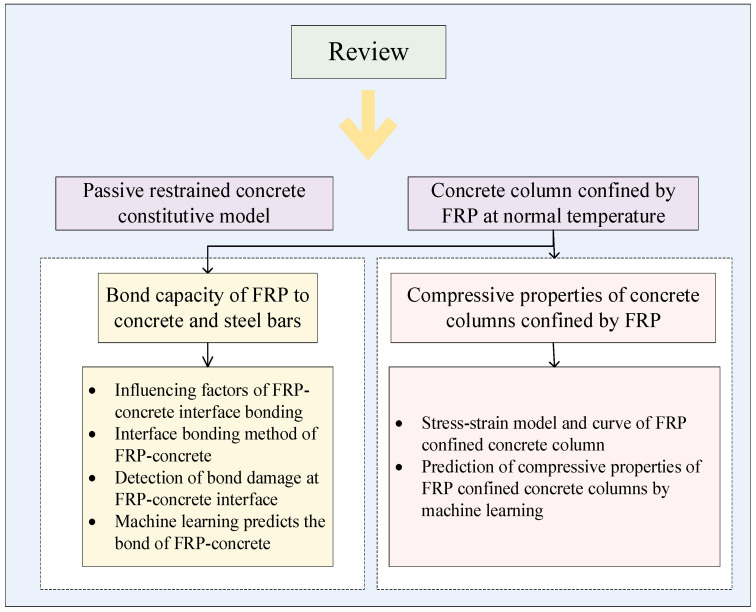
Overview idea diagram.

This paper aims to comprehensively review the effect of FRPs on the mechanical properties of concrete, An extensive literature search was conducted using databases, such as Web of Science, Scopus, Google Scholar, and Knowledge, selecting one hundred articles focused on FRP-confined concrete columns; the selection process prioritized studies offering both experimental and theoretical insights, and the study was analyzed based on the methodology, results, and relevance to current engineering practice. This paper aims to provide a general framework, in order to be supported by substantial data on influencing factors, and conducts a systematic review of the latest research findings to incorporate the latest research results, to explore the mechanical behavior of FRP-reinforced concrete at ambient temperatures and to propose the direction of the future research and the challenges of the future research, by summarizing and analyzing supported by substantial data on influencing factors, and it conducts a systematic review of the latest research findings. An overview of this paper is shown in Figure 3.

## 2. Passive Constrained Concrete Constitutive Model

Currently, the main forms of passively restrained concrete include hoop-restrained concrete, steel pipe concrete, FRP pipe-restrained concrete, FRP coil-restrained concrete, and other forms. In recent years, scholars have conducted numerous experimental and theoretical studies on different constrained forms of passively constrained concrete proposing various theoretical models for restrained concrete. Based on the assumptions underlying the models, the existing models are divided into two main categories.

(1) Assumption of constant restraining stresses

This type of model typically assumes that the strength of restrained concrete is solely dependent on the total restraining stress fr.

Mander et al. [19] conducted axial compression tests on 31 bar-restrained concrete columns with different cross-sectional shapes, including circular, square, and rectangular sections. Based on the reliable test results, Mander proposed a unified stress–strain model for restrained concrete. For circular cross-sections, the model introduces the *k_e_* confinement effect factor to calculate the total confined stress f*_r_*(Pa) as follows.

(1) $$f_{r}=\frac{1}{2}k_{e}\rho_{s}\sigma_{xy}$$

For steel hoop constraints:

(2) $$k_{e}=\frac{{\left(1-\frac{s^{\prime }}{2d_{s}}\right)}^{2}}{1-\rho_{cc}}$$

For spiral stirrup constraints:

(3) $$k_{e}=\frac{{\left(1-\frac{s^{\prime }}{2d_{s}}\right)}^{2}}{1-\rho_{cc}}$$

(4) $$\rho_{s}=\frac{A_{SP}\pi d_{s}}{\frac{\pi }{4}d_{s}^{2}s}=\frac{4A_{sp}}{d_{s}s}$$

where *k_e_* is the constraint effect coefficient, s is the center distance of the transverse rebar, σ_xy_ (Pa) is the stress of the concrete, s′ (m) is the edge distance between the transverse rebar, *ds* (m) is the center diameter of the hoop ring in the section of the member, ρs is the ratio of the steel bar volume to the confined concrete volume, *Asp* (m^2^) is the section area of the steel bar, and *ρcc* is the ratio of the longitudinal steel bar area to the core area of the section.

Mander’s model assumes that the confining stress provided by stirrups remains constant, primarily influenced by factors, such as the stirrup yield strength, spacing, volume proportion, and the relative size of the effective concrete area.

The fundamental formula for the stress–strain model of confined concrete is as follows:

(5) $$f_{cc}=\frac{f_{ccp}(\varepsilon /\varepsilon_{cc})r}{r-1+{\left(\varepsilon /\varepsilon_{cc}\right)}^{r}}$$

(6) $$r=\frac{E_{c}}{E_{c}-E_{\sec}}$$

(7) $$\varepsilon_{\mathrm{cc}}=\varepsilon_{co}\left[1+5(\frac{f_{ccp}}{f_{c}}-1)\right]$$

(8) $$E_{c}=5000\sqrt{f_{co}^{\prime }}$$

(9) $$E_{\sec}=\frac{f_{ccp}}{\varepsilon_{cc}}$$

where *f_co_* is the peak stress of unconstrained concrete, *ε_co_* is the corresponding strain, and *εco* is assumed to be 0.002. In this model, the five-parameter failure criterion proposed by Williaman and Warnke [20] is used to calculate the *f_ccp_* of confined concrete peak stress, and the relevant parameters are calculated based on Schickert and Winkler’s [21] triaxial test results. The calculation formula of *f_ccp_* is as follows:

(10) $$f_{ccp}=f_{co}^{\prime }(-1.254+2.254\sqrt{1+\frac{7.94f_{r}}{f_{co}^{\prime }}}-2\frac{f_{r}}{f_{co}^{\prime }})$$

**Figure 4:**
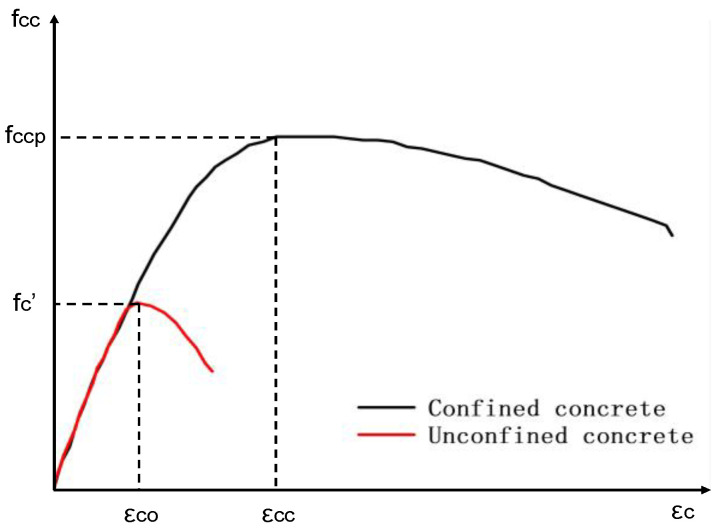
Mander’s stress–strain model of restrained concrete [19].

Mander’s stress–strain model for confined concrete is shown in Figure 4:

Susantha et al. [22] developed a stress–strain model for CFST (concrete-filled steel tube) columns with various section shapes. For circular CFST columns, Tang et al. [23] proposed a constrained stress calculation model that considers the evolving Poisson ratios of both concrete and steel tubes under column loading, utilizing an empirical factor *β*. Hence, the formula for calculating fr, the constrained stress in concrete filled steel tubes, is as follows:

(11) $$f_{r}=\beta_{s}\frac{2t}{D_{0}-2t}\sigma_{xy}$$

(12) $$\beta_{s}=v_{e}-v_{s,\max}=v_{e}-0.5$$

(13) $$v_{e}=0.2312+0.3582v_{e}^{\prime }-0.1524(\frac{f_{c}^{\prime }}{\sigma_{sy}})+4.843v_{e}^{\prime }(\frac{f_{c}^{\prime }}{\sigma_{sy}})-9.169{\left(\frac{f_{c}^{\prime }}{\sigma_{sy}}\right)}^{2}$$

(14) $$v_{e}^{\prime }=\frac{0.881}{10^{6}}{\left(\frac{D_{0}}{t}\right)}^{3}-\frac{2.58}{10^{4}}{\left(\frac{D_{0}}{t}\right)}^{2}+\frac{1.953}{10^{2}}(\frac{D_{0}}{t})+0.4011$$

where *ve* and *vs* represent the maximum Poisson ratio of the steel pipe with and without filled concrete, respectively, and *vs* is 0.50 at the maximum strength point. *Ve*′ is an empirical formula. This equation applies to components with (*fc*′/*σsy*) values in the range of 0.04 to 0.20. It can be seen that the size of *fr* mainly depends on the yield strength, ratio of the diameter to the thickness of the steel pipe, and concrete strength. To study the constitutive relationship of confined concrete, Susantha et al. adopted Mander’s stress–strain model for peak stress determination. The following formula is used:

(15) $$f_{ccp}=f_{c}^{\prime }+4f_{r}$$

Li Bing et al. [24] performed axial compression tests on high-strength concrete columns reinforced with hoops, employing both ordinary strength and ultra-high-strength steels, and conducted tests using concrete of varying strengths, from ordinary to high-strength. Based on their experimental findings, they formulated a stress–strain model specifically designed for confined high-strength concrete. They utilized Mander’s model to calculate fr. Regarding the stress–strain model for confined concrete, Li Bing et al. segmented the stress–strain curve into four distinct segments based on specific key points. They adjusted the curve formula to better replicate the actual behavior. Consequently, they modified the model proposed by Muguruma and Watanabe [25], resulting in a new constrained concrete stress–strain model.

(16) $$\begin{array}{c} f_{cc}=E_{C}\varepsilon_{z}+\frac{\left(f_{c}^{\prime }-E_{c}\varepsilon_{co}\right)}{\varepsilon_{co}^{2}}\varepsilon_{z}^{2}\;\;\;\;\;\;\;\;\;\;\;\;\;\;\;\;\;\;\;\;\;0\leq \varepsilon_{z}\leq \varepsilon_{co}\\ f_{cc}=f_{ccp}-\frac{\left(f_{ccp}-f_{c}^{\prime }\right)}{{\left(\varepsilon_{cc}-\varepsilon_{co}\right)}^{2}}\times {\left(\varepsilon_{z}-\varepsilon_{cc}\right)}^{2}\;\;\;\;\;\;\;\;\;\varepsilon_{co}\leq \varepsilon_{z}\leq \varepsilon_{cc}\\ f_{ccp}-\beta \frac{f_{ccp}}{\varepsilon_{cc}}\times \left(\varepsilon_{z}-\varepsilon_{cc}\right)\geq 0.4f_{ccp}\;\;\;\;\;\;\;\varepsilon_{z}\geq \varepsilon_{cc} \end{array}$$

Among these, *β* is a parameter that controls the slope of the post-peak descending stage of the stress–strain curve, and its value is dependent on the material strength. Specifically, for ordinary strength stirrup-confined concrete, *β* is set to 0.2 when *fc′* is less than or equal to 80 MPa; otherwise, *β* is 0.08.

It is important to note that in the post-peak stage of the stress–strain curve, the response remains linear until the confined concrete stress decreases to 0.4 times *f_ccp_* (peak stress), after which the curve transitions to a stage of residual stress that remains constant until the concrete reaches its ultimate strain *εcu*.

For calculating the peak stress *f_ccp_* of confined concrete, Li Bing et al. adopted a five-parameter failure criterion similar to the Mander model. They used triaxial test results from Khaloo and Ahmad [26] specifically for high-strength concrete, enabling them to derive a more precise calculation formula for the *f_ccp_* of confined high-strength concrete and its corresponding strain *εcc*.

(17) $$f_{ccp}=f_{c}^{\prime }\left[-0.413+1.413\sqrt{1+11.4\frac{f_{r}}{f_{c}^{\prime }}}-2\frac{f_{r}}{f_{c}^{\prime }}\right]$$

For common strength stirrup constraints:

(18) $$\frac{\varepsilon_{\mathrm{cc}}}{\varepsilon_{co}}=1+384{\left(\frac{f_{r}}{f_{c}^{\prime }}\right)}^{2}$$

For ultra-high strength stirrup constraints:

(19) $$\begin{array}{c} \frac{\varepsilon_{cc}}{\varepsilon_{co}}=1.0+\left(120-1.554f_{ccp}\right){\left(\frac{f_{r}}{f_{c}^{\prime }}\right)}^{2}\;\;\;\;f_{c}^{\prime }\leq 50MP\\ \frac{\varepsilon_{cc}}{\varepsilon_{co}}=1.0+\left(71.4-0.23f_{ccp}\right){\left(\frac{f_{r}}{f_{c}^{\prime }}\right)}^{2}\;\;\;\;f_{c}^{\prime }>50MP \end{array}$$

Elremaily and Azizinamini [27] derived a formula relating fr, the confined concrete stress, to the circumferential stress *σ_sθ_* based on a free body force diagram. Through the linear fitting of experimental data, they determined that the circumferential strain *σ_sθ_* equals 0.1 times the yield stress of steel (*σsy*) within the slenderness ratio range of 9.06 to 26.02.

(20) $$f_{r}=-\frac{2\sigma_{s\theta }t}{D_{0}-2t}$$

The constrained concrete stress–strain model similarly adopts Mander’s model. Regarding the constitutive model of steel pipe, Elremaily and Azizinamini omit the influence of the steel pipe thickness and emphasize the interaction between the concrete and steel pipe. They propose that the steel pipe experiences plane stress with annular tension and longitudinal compression, assuming a linear elastic-ideal elastic–plastic stress–strain relationship. The Von Mises yield criterion is employed to define the yield surface of the steel pipe.

(21) $$\sigma_{v}^{2}-\sigma_{\theta }\sigma_{v}+\sigma_{\theta }^{2}=\sigma_{xy}$$

Zhang et al. [28] introduced the steel tube constraint index *ξs* into their proposed FRP constrained concrete filled steel tube constitutive model. The specific formula is as follows:

(22) $$\xi_{s}=\frac{A_{s}\sigma_{sy}}{A_{c}f_{c}}$$

Considering the contribution of FRP coils and steel tubes to the bearing capacity of the member, Zhang utilized Jiang et al.’s [29] ultimate strain model for FRP-confined concrete. The formula for calculating the constrained stress flr provided by FRP is as follows:

(23) $$f_{\mathrm{tr}}=\frac{2E_{f}\varepsilon_{f}t_{f}}{D_{0}}$$

It can be seen that the determination of *ftr* is only related to the elastic modulus *Ef* of FRP, 0.58 times the ultimate tensile strain *εf* of FRP, the thickness *tf* of FRP, and the external diameter Do of the member.

For steel tubes, Zhang used the steel tube constraint index to consider the constraint effect and divided the stress–strain model of FRP-bound concrete-filled steel tube members into two stages: before and after FRP failure. When FRP is involved in the work:

(24) $$\frac{f_{cu}}{f_{c}^{\prime }}=1+1.27\xi_{s}+1.28\xi_{f}ac$$

(25) $$\frac{\varepsilon_{\mathrm{cu}}}{\varepsilon_{co}}=1+1.75{\left(\frac{f_{r}}{f_{c}^{\prime }}\right)}^{17.2}+\xi_{s}$$

(26) $$\frac{f_{c}^{\prime }}{f_{cc}^{\prime }}=\frac{x\cdot a}{a-1+{\left(x\right)}^{a{\left(x+\delta \right)}^{b}+c}}$$

(27) $$\begin{array}{l} c=\frac{\ln(\frac{f_{cc}^{\prime }\varepsilon_{cu}a}{f_{cc}^{\prime }\varepsilon_{cc}^{\prime }}-a+1)}{\ln\varepsilon_{cu}-\ln\varepsilon_{cc}}-a{\left(\frac{\varepsilon_{cu}}{\varepsilon_{cc}^{\prime }}+\delta \right)}^{-0.1} \end{array}$$

where *x* = *εc/εcc*, *a* = *Ecs/(ECS − ESEC)*, *Esec* = *fcc/εc*. The value of *b* is 0.1, and the value of *δ* is −0.1. *f_cu_* and *εcu* are the ultimate stress and corresponding strain of the member, and *fcc*′ and *εcc* are the stress and strain corresponding to the turning point from the nonlinear transition region to the linear strengthening region in the stress–strain curve of the member.

When FRP fails, follow the model proposed by Popovics [30] and let *b* = *c* = 0, thus obtaining the calculation model of *fc*:

(28) $$\frac{f_{c}}{f_{cc}}=\frac{x\cdot a}{a-1+{\left(x\right)}^{a}}$$

This model assumes that the total confining stress *f_r_* remains constant during loading, and is solely dependent on material properties and the member’s diameter-to-thickness ratio, without considering the influence of circumferential and longitudinal strains. However, in passively restrained concrete members subjected to compression, the interaction between the restrained concrete and the steel pipe/FRP (tube) becomes significant. As the longitudinal strain increases, cracking occurs in the concrete, which leads to an increase in circumferential strain. This interaction between the concrete and the restraining material causes the confining stress to vary with the circumferential strain throughout the loading process. Predictions of the mechanical properties of members, particularly those using high-strength materials or large diameter-to-thickness ratios, will likely result in significant errors if based on this assumption.

(2) It is assumed that the constraint stress is related to the loading history.

In predicting the strength or stress of confined concrete, these models take into account that the confined stress varies over the entire loading process in response to circumferential and longitudinal strains. These models are generally categorized into two main types: design models and analysis models.

1. Design model.

This type of model is developed based on empirical formulas obtained from a regression analysis of experimental data. Consequently, the accuracy of these models is highly dependent on the reliability and comprehensiveness of the experimental database. For example, Xiao and Wu [31] proposed a relatively straightforward bilinear stress–strain model for confined concrete based on the findings from 27 axial compression tests of FRP confined concrete.

In the initial phase, when the confined concrete stress *fcc* is less than a certain critical stress *fc*′, the stress–strain model is derived from elastic theory:

(29) $$f_{cc}=E_{c}\varepsilon_{z}+2v_{c}f_{r}$$

(30) $$\varepsilon_{\theta }=-\frac{v_{c}}{1+\frac{C_{j}}{E_{c}}(1-v_{c}-2v_{c}^{2})}\varepsilon_{z}$$

(31) $$f_{r}=-C_{j}\varepsilon_{\theta }$$

(32) $$C_{j}=\frac{2t}{D_{0}}E_{j}$$

When *fcc ≥ fc*′:

(33) $$f_{cc}=\alpha f_{c}^{\prime }+kf_{r}$$

(34) $$k=4.1-0.75\frac{f_{c}^{\prime }}{C_{j}}$$

(35) $$\varepsilon_{\theta }=\varepsilon_{\mathrm{ro}}^{\prime }-v_{c}^{\prime }\varepsilon_{z}$$

(36) $$v_{c}^{\prime }=7{\left(\frac{f_{c}^{\prime }}{C_{j}}\right)}^{0.8}$$

where *Cj* is the constraint modulus and *Ej* is the tensile modulus of the FRP. The average value of coefficient *α* is 1.10 based on the test data, and the calculation formula of coefficient *k* is obtained through z regression analysis. *εro*′ is the intercept at *εz* = 0, and the mean value of *εro*′ based on the test results is −0.0005.

The model described above is derived from a regression analysis of experimental data and is based on elasticity theory. The accuracy and applicability of this model primarily depend on the reliability of the test data, the comprehensiveness of the parameters, and the rationality of the selected model type. Consequently, the development and validation of such a model necessitate a substantial amount of experimental data.

Although the bilinear model is simple and efficient for predicting the stress–strain relationship of confined concrete, it notably deviates from the actual stress–strain curve, particularly lacking a transitional phase. Therefore, the rationality of the model requires further validation. As a result, this model encounters difficulties in accurately predicting the real stress–strain behavior of FRP-confined concrete.

Moreover, since this model only conducts regression analysis on the test data of FRP-confined concrete members, it is not applicable to other types of confined concrete.

2. Analytical model.

In this type of model, it is recognized that the strength of confined concrete is not only closely tied to *fr*, but also that *fr* varies continuously during loading due to the interaction between the concrete and the surrounding confining materials. This model not only investigates the stress–strain relationship between the core concrete and the confining material but also examines their interaction under conditions of transverse deformation coordination.

Therefore, the key aspect of the model lies in accurately simulating the relationship between the core concrete and the confining material, particularly by accurately modeling the circumferential strain. Therefore, the key aspect of the model lies in accurately simulating the relationship between the core concrete and the confining material, particularly by accurately modeling the circumferential strain.

➀ The assumption that the stress–strain relationship of confined concrete is independent of the applied stress path.

Building on the assumption that the stress–strain relationship of restrained concrete is unaffected by the restraining stress path, Jiang and Teng [29] proposed an analytical model for FRP coil restrained concrete. This model assumes that the longitudinal stress of actively restrained concrete can directly represent the longitudinal stress of passively restrained concrete under the same restraining stress and longitudinal strain. Additionally, Johansson developed an analytical model for concrete confined by steel pipes, which is also based on the interaction between the core concrete and the steel pipe. In this study, the stress–strain model for restrained concrete was adapted from the active restrained concrete model proposed by Attard and Setunge [32], which is based on standard triaxial tests and can be applied to a broader range of concrete strengths (20–130 MPa) and restrained stresses (1–20 MPa) than other active models. The original expression for the curve was proposed by Sargin [33]:

(37) $$Y=\frac{AX+BX^{2}}{1+CX+DX^{2}}$$

In the formula, *Y = f_cc_/f_ccp_*, *X = εz/εcc*. A, B, C, and D are empirical constants, and *C = A − 2*, *D = B + 1*. Two sets of empirical constants need to be taken for the rising stage and the falling stage of the curve, respectively, so the stress–strain model of the confined concrete is as follows:

(38) $$\frac{f_{cc}}{f_{ccp}}=\frac{A(\varepsilon_{z}/\varepsilon_{cc})+B{\left(\varepsilon_{z}/\varepsilon_{cc}\right)}^{2}}{1+(A-2)(\varepsilon_{z}/\varepsilon_{cc})+(B+1){\left(\varepsilon_{z}/\varepsilon_{cc}\right)}^{2}}$$

The formula for calculating the strength of confined concrete *f_ccp_* and its corresponding strain *εcc* is as follows:

(39) $$f_{ccp}=f_{c}^{\prime }{\left(\frac{f_{r}}{0.56\sqrt{f_{c}^{\prime }}}\right)}^{1.25(1+0.062\frac{f_{r}}{f_{c}^{\prime }})f_{c}^{\prime -0.21}}$$

(40) $$\varepsilon_{\mathrm{cc}}=\varepsilon_{cu}\left[1+(17-0.06f_{c}^{\prime })\frac{f_{r}}{f_{c}^{\prime }}\right]$$

When *εz < εcc*:

(41) $$A=\frac{E_{c}\varepsilon_{cc}}{f_{ccp}}$$

(42) $$B=\frac{(A-1{)}^{2}}{0.55}-1$$

When *εz ≥ εcc*:

(43) $$A=(\frac{\varepsilon_{2i}-\varepsilon_{i}}{\varepsilon_{cc}})\left[\frac{\varepsilon_{2i}f_{i}}{\varepsilon_{i}(f_{ccp}-f_{i})}-\frac{4\varepsilon_{i}f_{2i}}{\varepsilon_{2i}(f_{ccp}-f_{2i})}\right]$$

(44) $$B=(\varepsilon_{i}-\varepsilon_{2i})\left[\frac{\varepsilon_{2i}f_{i}}{\varepsilon_{i}(f_{ccp}-f_{i})}-\frac{4f_{2i}}{\varepsilon_{2i}(f_{ccp}-f_{2i})}\right]$$

(45) $$\varepsilon_{i}=\varepsilon_{cc}\left[2+\frac{2.5-0.3\ln(f_{c}^{\prime })-2}{1+1.12{\left(f_{r}/f_{c}^{\prime }\right)}^{0.26}}\right]$$

(46) $$\varepsilon_{2i}=2\varepsilon_{i}-\varepsilon_{cc}$$

(47) $$f_{i}=f_{ccp}\left[1+\frac{1.41-0.17\ln(f_{c}^{\prime })-1}{1+5.06{\left(f_{r}/f_{c}^{\prime }\right)}^{0.57}}\right]$$

(48) $$f_{2i}=f_{ccp}\left[1+\frac{1.45-0.25\ln(f_{c}^{\prime })-1}{1+6.35{\left(f_{r}/f_{c}^{\prime }\right)}^{0.62}}\right]$$

where, *εi*, *fi* refer to the strain and stress at the first inflection point of the curve, and *εi*, *fi* corresponds to the strain and stress at the second inflection point of the descending section of the curve. To account for the interaction between concrete and the steel pipe, the volumetric strain model proposed by Imran and Pantazopoulou’s [34] volumetric strain model is adopted as the lateral strain model for concrete. They conducted triaxial tests on concrete cylinders and proposed a model where the relationship between the concrete volume strain *εvol* and longitudinal strain *εz* is initially linear. As the loading progresses, concrete develops splitting cracks that gradually accelerate transverse deformation, which surpasses longitudinal deformation, thereby leading to a nonlinear relationship between *εvol* and *εz*. In the elastic stage, the model assumes that concrete exhibits isotropic and elastic behavior, allowing for the prediction of lateral deformation based on generalized Hooke’s law. However, once concrete cracks, it becomes anisotropic and non-uniform, necessitating different values for Poisson’s ratio and the elastic modulus in different directions compared to the elastic stage. Since an accurate prediction of transverse deformation is essential for this model, simulating the longitudinal load-deformation curve of concrete-filled steel tube (CFST) columns becomes challenging.

This model assumes that the confining stress (*fr*) evolves with the development of circumferential strain in confined concrete and that the stress–strain relationship of confined concrete remains independent of the confining stress path. Therefore, an active confined concrete constitutive model can directly simulate the stress–strain curve of passively confined concrete. The changing confining stress in passively confined concrete is represented through incremental iteration. However, recent studies have explored the impact of stress paths on the stress–strain relationship of passively confined concrete. Scholars have found that the confined stress path significantly impacts this relationship. Assuming independence of the stress path when studying the stress–strain relationship of passively confined concrete can lead to significant errors.

② The stress–strain behavior of confined concrete is assumed to correlate with the constrained stress path.

This kind of model holds that the longitudinal stress of confined concrete is affected by the stress path, and the effect of the confined stress path should be considered when predicting the stress–strain curve of passively confined concrete. Based on the constrained concrete constitutive model proposed by other scholars and 346 test results, Chen et al. [35] compared the stress–strain relationship of concrete under active confinement and FRP confinement and established a constrained concrete analysis model considering stress paths to predict the mechanical properties of FRP-coiled concrete columns or CFST columns. The active constrained concrete model proposed by Popovics is adopted and the model proposed by other scholars is combined to obtain the calculation formulas of *f_ccp_* and *ε_cc_*. The specific details are as follows:

(49) $$\frac{f_{cc}}{f_{ccp}}=\frac{(\varepsilon_{z}/\varepsilon_{cc})\left[E_{c}/(E_{c}-f_{ccp}/\varepsilon_{cc})\right]}{\left[E_{c}/(E_{c}-f_{ccp}/\varepsilon_{cc})\right]-1+{\left(\varepsilon_{z}/\varepsilon_{cc}\right)}^{\left[E_{c}/(E_{c}-f_{ccp}/\varepsilon_{cc})\right]}}$$

(50) $$E_{c}=4700\sqrt{f_{c}^{\prime }}$$

(51) $$f_{ccp}=f_{c}^{\prime }+6.7f_{r}^{0.83}$$

(52) $$\frac{\varepsilon_{\mathrm{cc}}}{\varepsilon_{co}}=1+17.5\frac{f_{r}}{f_{c}^{\prime }}$$

Chen employs the explicit equation for the lateral strain model of confined concrete proposed by Teng et al.:

(53) $$\frac{\varepsilon_{c}}{\varepsilon_{co}}=0.85(8\frac{f_{r}}{f_{c}^{\prime }}+1)\left(\right.{\left\{1+0.75(\frac{-\varepsilon_{\theta }}{\varepsilon_{co}})\right\}}^{0.7}-\exp\left[-7(\frac{-\varepsilon_{\theta }}{\varepsilon_{co}})\right]\left.\right)$$

Furthermore, Chen investigated the influence of the constrained stress path by comparing the annular longitudinal strain curve and longitudinal stress–strain curve of both actively constrained and FRP-constrained concrete. He also explored the ratio of longitudinal stresses under passive and active constraints, considering the material’s constraint stiffness, constrained stresses, and concrete strength. The results were obtained by fitting an analytical formula for the safety factor *β* (*β* ≤ 1), where fcc,active = β · fcc, active, to examine the impact of the constrained stress path on the longitudinal stress of restrained concrete. However, the cyclic strain formula was derived from only 13 experimental data points, and its accuracy requires validation through additional data. Recent studies have corroborated Chen’s conclusions, showing that the stress–strain relationship of restrained concrete is influenced by the constrained stress path. These studies, both experimental and theoretical, have focused on the stress paths of restrained concrete. Lim and Ozbakkaloglu (2015) [36] conducted axial compression tests on 63 FRP-constrained and actively stress-constrained normal-strength and high-strength concrete columns. They found that the development of passively constrained stresses did not precisely follow the path of actively constrained stresses. Li and Wu (2016) [37] performed monotonic and cyclic axial compression tests, concluding that the stress path had a minimal effect on the overall performance of FRP-restrained concrete under monotonic axial compression. However, this conclusion does not hold for restrained concrete columns subjected to large volumetric strains under cyclic axial loading. Xiong et al. (2016) [38] proposed a new stress–strain model for actively restrained concrete, validated by comparing the model’s predictions with the load-deflection test curves of actively restrained concrete. Wu and Cao (2017) [39] tested 55 specimens under various loading paths and concluded that the stress–strain relationship of FRP-restrained concrete was significantly influenced by the restrained stress path. Lin et al. (2018) [40] concluded from the test results of 18 axially loaded CFST columns that the longitudinal stresses of restrained concrete were correlated with the restrained stress paths under weak confining effects. However, with the increase in the confinement effect, the influence of the confining stress path on the confined concrete stresses was not significant. Chang (2023) [41] proposed a strain-path-dependent stress–strain model for UHPC restrained by hoop and fiber constraints by using a database of 10 sets of UHPC column test results to alleviate the drawbacks of design-oriented models. Chen (2024) [42] fabricated six specimens for axial compression load testing and finite element parametric analysis. The results showed that the coral aggregate seawater sea sand concrete (CSSC) was in triaxial compression due to the polyvinyl chloride (PVC), which improved the integrity of the CFRP, and the PVC pipe provided a stress transfer path to the CFRP.

The discussion above demonstrates that the restrained stress path significantly influences both the passive restraining stress and the longitudinal stress–strain relationship of concrete under axial compression. This occurs because the restraining stress in actively restrained concrete is artificially maintained as constant throughout the loading process. The early application of restraining stress delays crack formation and propagation, resulting in higher stiffness under the same restraining stress, allowing the actively restrained concrete to withstand larger longitudinal loads. In contrast, the development of confining stress in passively confined concrete varies with crack progression. Consequently, to achieve the same longitudinal strain under equivalent restraining stress, a greater load must be applied to actively restrained concrete, resulting in higher longitudinal stress. Additionally, different forms of restraint influence the crack development patterns in restrained concrete. To accurately predict the stress–strain curves of FRP-restrained concrete columns, investigate the mechanical properties of these members, and ensure their reliable application in practical projects, it is essential to fully understand the stress–strain relationship, consider the effects of restrained stress paths, and accurately model the interaction between concrete columns and FRP.

## 3. FRP Restrained Concrete Columns at Ambient Temperature

### 3.1. Bonding Ability of FRP to Concrete and Steel Reinforcement

Interfacial bonding is the primary failure mode of FRP, and most studies have focused on interfacial bonding damage. Other research has addressed rupture, shear damage, concrete compression failure, and concrete cover separation.

#### 3.1.1. Factors Affecting FRP-Constrained Concrete Interfacial Bonding

**Figure 5:**
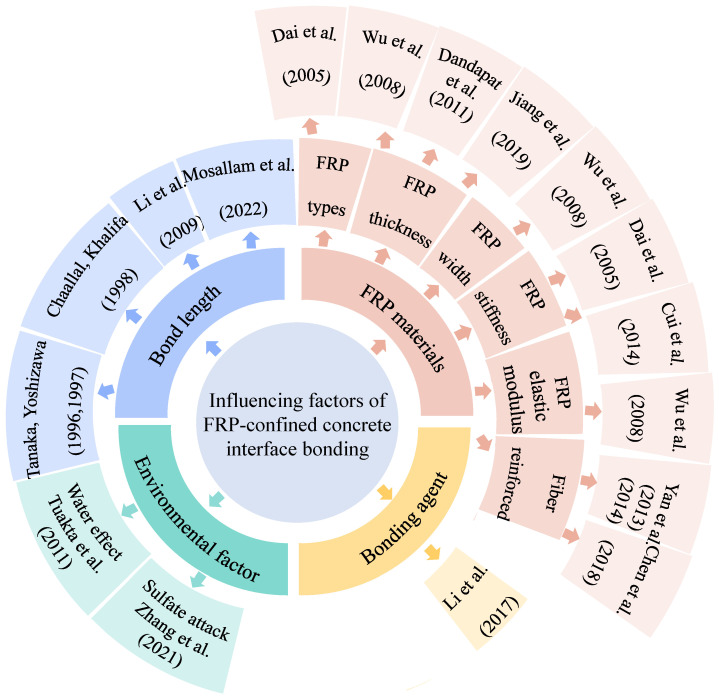
Factors affecting FRP-constrained concrete interfacial bonding [43,44,45,46,47,48,49,50,51,52,53,54,55,56,57,58,59].

Numerous studies have explored the factors affecting the interfacial bond strength between FRP and concrete, with the influencing factors and relevant studies summarized in Figure 5. Tanaka and Yoshizawa (1996, 1997) [43,44] proposed a method to calculate the average interfacial bond stress; however, the prediction accuracy is limited due to the neglect of FRP bond length. Chaallal and Khalifa (1998) [45,46] experimentally demonstrated that bond length significantly affects the strength of the FRP-constrained concrete interface once a certain threshold is exceeded. Dai et al. (2005) [47] proposed a mathematical method incorporating different FRP stiffnesses, materials, and adhesives. The method requires only two key parameters: interfacial fracture energy and ductility index, significantly improving the prediction accuracy of bond capacity.

Wu et al. (2008) [48] found experimentally that the FRP-constrained concrete interfacial bonding capacity is closely related to the behavior of concrete and is affected by the width, thickness, and elastic modulus of the FRP sheet. Li et al. (2009) [49] simulated the FRP-constrained concrete interface behavior under seismic conditions and found that FRP at the joints was prone to debonding. Niroomandi et al. (2010) [60] demonstrated that FRP-reinforced concrete frame joints can significantly enhance their ductility.

Dandapat et al. (2011) [50] investigated the damage patterns in FRP-restrained circular concrete columns, highlighting that poor bonding due to high shear stresses becomes more problematic as the fabric thickness and strength increase. Tuakta et al. (2011) [51] investigated the effect of moisture on the FRP-constrained concrete bond strength and proposed a model to predict bond strength based on moisture variation.

Yan et al. (2013, 2014) [52,53] experimentally demonstrated that the interfacial bond strength of coir and flax-fiber-reinforced composites is strongly correlated with their confinement properties. Cui et al. (2014) [54] experimentally investigated the bond behavior of FRP steel wire sheathing-restrained concrete and found that radial strain plays a crucial role in enhancing bond strength. Li et al. (2017) [55] developed a modified epoxy resin adhesive that significantly improved the bonding performance between FRP and concrete.

Chen et al. (2018) [56] investigated the effect of a flax-fiber-reinforced FRP on coconut fiber-reinforced concrete composites. Their results demonstrated that the enhanced mechanically bonded interface significantly improved the bond between the polymer and the concrete core. Jiang et al. (2019) [57] found that the relationship between the interfacial bond strength and plastic hinge length exhibited a complex trend as the FRP content increased, although changes in bonding conditions did not significantly affect the response of FRP-constrained RC columns.

Saeed et al. (2020) [61] demonstrated that bond strength is closely related to variations in the burial length and hole diameter by studying FRP anchors reinforcing concrete columns. Zhang et al. (2021) [58] analyzed the damage mode of the FRP-constrained concrete interface under sustained loading and sulfate erosion, proposing an interfacial bond-slip model that accounts for combined effects.

Recently, Mosallam et al. (2022) [59] evaluated the bonding behavior at the CFRP-constrained concrete interface using the near-surface mounting (NSM) technique. They found that increasing the bonding layer length enhances the ultimate failure load and fracture strain of the CFRP.

#### 3.1.2. FRP-Constrained Concrete Interfacial Bonding Methods

The choice of the interfacial bonding method significantly influences the reinforcement performance of FRP-constrained concrete columns. Deb et al. (2010) [62] demonstrated that the type of interfacial bond directly affects the ultimate strength of concrete columns. Moshiri et al. (2015) [63] investigated four bonding methods and found that the grooving method (GM) significantly delayed CFRP buckling, thereby enhancing the load-bearing capacity of RC columns. Nossoni et al. (2015) [64] compared bonded and unbonded fiber concrete, showing that unbonded concrete mitigated the stress concentration effect but accelerated reinforcement corrosion.

Torabian et al. (2017) [65] investigated the reinforcement effect of GM compared to conventional bonding methods under eccentric loading, revealing that GM was more effective in delaying FRP buckling and increasing the strength. Saljoughian et al. (2018) [66] experimentally demonstrated that the GM technique significantly improves the compressive strength and ductility of concrete columns under cyclic axial compression. Saljoughian et al. (2020) [67] further demonstrated that combining two GM grooving methods (EBROG and EBRIG) effectively prevents the debonding and buckling of CFRP panels.

Mostofinejad et al. (2021) [68] found that the EBROG method significantly increased the load-carrying capacity under eccentric loading when compared to other bonding methods. Taherirani et al. (2022) [69] analyzed the advantages of the EBROG method under eccentric loading using Particle Image Velocimetry (PIV). Ghaleh et al. (2022) [70] evaluated the GM in the EBRIG technique for the first time, with experimental results showing that the EBRIG joints outperformed the EBROG joints in terms of performance.

Due to the significant influence of interfacial bonding methods on FRP-restrained concrete columns, several scholars have recently reviewed these bonding methods [70,71].

#### 3.1.3. Detection of Bond Damage at the FRP-Constrained Concrete Interface

Numerous studies have been conducted on bond damage monitoring at the FRP-constrained concrete interface. Ghosh (2011) [72] employed infrared thermography to quantitatively monitor the onset and progression of damage at the FRP system and the FRP-constrained concrete interface. Based on the thermal intensity, the damage can be categorized into types, such as composite internal interlayer debonding or composite-concrete interface debonding, and the severity of the damage is quantitatively monitored.

In recent years, Wang et al. (2019) [73] employed a wave-based method using piezoelectric ceramics (PZT) to detect damage at the CFRP-constrained concrete interface. The results demonstrated that this method effectively tracked the development of damage over time. Zhu et al. (2019) [74] developed a finite element model of FRP-RC columns under eccentric loading, based on previous studies. They simulated bond-slip behavior by introducing a spring element between the FRP reinforcement and the concrete. It was found that the initial stiffness of the bond-slip was the most sensitive parameter affecting the compressive bending performance of the columns.

Deng et al. (2021) [75] utilized a PZT-based electromechanical impedance (EMI) method to detect damage at the CFRP-constrained concrete interface. Their study showed that the EMI method was more sensitive to initial concrete damage and debonding phenomena compared to wave-based methods. Kim et al. (2021) [76] employed various destructive techniques to measure bonding capacity, and although these methods provided valuable data, they were costly and posed safety risks, limiting their practical application.

Despite these advancements, significant gaps remain in the current research on bond damage detection at the FRP-constrained concrete interface. Further in-depth exploration and participation from more scholars are needed to promote progress in this area.

#### 3.1.4. Machine Learning to Predict FRP-Constrained Concrete Bonds

Subsequently, many researchers have developed new models to improve prediction accuracy. Although these models offer some improvements over earlier approaches, they are less effective when used with other data, and traditional prediction methods cannot account for multiple factors simultaneously. Moreover, experimental data remain limited. To address these issues, researchers have turned to more sophisticated, data-driven methods to model the FRP-constrained concrete interfacial bonding capacity. Machine learning (ML)-based methods, in particular, have emerged as promising alternatives due to their adaptive learning capabilities, robustness, and ability to model nonlinear and multivariate relationships. By utilizing experimental datasets, ML models can be trained to develop faster, more reliable, and cost-effective tools for calculating cohesive bonding. Furthermore, by incorporating larger and more diverse datasets during training, ML models can achieve a higher level of generalization, enhancing their applicability across different scenarios and conditions.

These ML methods have made significant strides in predicting and understanding the complex interfacial bonding behavior between FRP fibers and concrete in real-world engineering applications. Among the ML algorithms, artificial neural networks (ANNs) have been widely used to predict the interfacial bonding capacity between FRP and concrete. However, other ML algorithms may offer superior accuracy and performance in this field.

Mashrei et al. (2013) [77] proposed a back-propagation neural network (BPNN) to simulate the bonding ability of FRP to concrete using 150 test data samples. Golafshani et al. (2015) [78] proposed a back-propagation neural network (BPNN) to simulate the bonding ability of FRP to concrete using 150 test data samples. Coelho et al. (2016) [79] proposed a back-propagation neural network (BPNN) to simulate the bonding ability of FRP to concrete using 150 test data samples. Köroğlu et al. (2019) [80] improved the accuracy of their bond strength predictions by modifying the ANN model.

Wang et al. (2012) [81] and Saghi et al. (2019) [82] used GEP to simulate the bonding ability of FRP to concrete. They found that GEP outperformed ANN in predicting the bond strength of FRP-constrained concrete composites. Gao et al. (2020) [83] proposed two hybrid models, ICA-ANN and ABC-ANN (Imperial Competitive Algorithm vs. Artificial Neural Network, Artificial Bee Colony Algorithm with Artificial Neural Network) and found that ICA-ANN model outperforms the ABC-ANN model for bond strength prediction.

Zhang et al. (2021) [84] collected data from 145 direct pullout tests and developed GEP and random forest (RF) models. The results showed that the GEP model had better prediction performance than some existing empirical models, with a lower mean absolute error (MAE), relative root mean square error (RMSE), mean absolute percentage error (MAPE), and integral absolute error (IAE). This study also found that the bond strength increased with the bond length, FRP axial stiffness, depth-to-width ratio, and concrete compressive strength, while the epoxy tensile strength had a smaller effect on the bond strength. In contrast, Cascardi et al. (2021) [85] predicted the debonding force of FRP panels bonded with epoxy to a concrete substrate using an ANN model.

Wang et al. (2022) [86] predicted the debonding force of FRP panels bonded with epoxy to a concrete substrate using an ANN model. Barkhordari et al. (2022) [87] employed several hybrid models to predict the FRP-constrained concrete interfacial bond and found that the RUN-ANN algorithm offered the highest prediction accuracy, with a coefficient of determination (R^2^) of 92% and the smallest error (0.078). Amin et al. (2022) [88] developed a mathematical relationship to estimate the interfacial bond strength of FRP laminates on notched concrete prisms using the GEP model. The genetic variance showed an optimal combination of 30 chromosomes, 11 head sizes, and 4 genes.

Alabdullh et al. (2022) [89] constructed a hybrid inheritance (HENS) model to predict the FRP-constrained concrete interfacial bond strength, and the experimental results demonstrated that the model effectively addressed the overfitting issue commonly encountered in CML models. Amin et al. (2022) [90] employed three integrated methods to predict the FRP-constrained concrete interfacial bond strength, finding that the light-gradient boosting machine (LIGHT GBM) was a reliable ML technique for predicting the bond strength of FRP laminates to concrete prisms.

The specific parameters and training details for these models are summarized in Table 2.

As can be seen from Table 2, different scholars use different machine learning models to predict the bonding ability of FRP and concrete columns. According to the research results, BPNN, ANN, SVR, GEP, RBFNN, LSVR, GEP and RF have advantages and disadvantages. The number of training sets, test sets, and hidden nodes has a great impact on the accuracy of machine learning models.

### 3.2. Compressive Performance of FRP-Restrained Concrete Columns

#### 3.2.1. Stress–Strain Model and Curves for FRP-Restrained Concrete Columns

In the past five years, significant progress has been made in optimizing stress–strain models for FRP-constrained concrete columns, including those using AFRP [91], BFRP [92,93,94,95], CFRP [96,97,98,99,100], and GFRP [101] for cylindrical and square columns. Yeou-Fong Li et al. [91] developed an intrinsic model to simulate the stress–strain behavior of AFRP-constrained concrete under uniaxial compression. They utilized the Mohr–Coulomb damage envelope theory to model the behavior of circular and square concrete columns constrained with varying layers of the AFRP. By conducting a regression analysis, they determined the corresponding axial strains under different conditions. Their constitutive model effectively predicted the maximum compressive strength of AFRP-constrained concrete columns.

Building on this work, Huang et al. [92] proposed enhanced strength and ultimate strain models for BFRP-constrained circular and square columns based on experimental data from several existing studies. These models improve upon previous predictions, offering a more accurate representation of the performance of BFRP-constrained concrete columns under axial load.

To address the gap in models for partially FRP-constrained concrete, Yang et al. [102] introduced a new stress–strain model for this case. The model makes several key assumptions:

(1) The stress–strain relationship can be categorized into strain-hardening type (Type 1) and strain-softening type (Type 2), and the curves with falling branches after the peak can be further categorized into Type 2-1 (fcu′ > fco′) and Type 2-2 (fcu′ < fco′);
(2) Both types of stress–strain curves consist of three segments, i.e., two linear curves connected by transition branches;
(3) The slope of the first line segment is equal to the modulus of elasticity of unconfined concrete, and the endpoint of the first line segment is (εo, 0.7fco′);
(4) The slope of the third linear branch is directly determined by the two endpoints.

**Figure 6:**
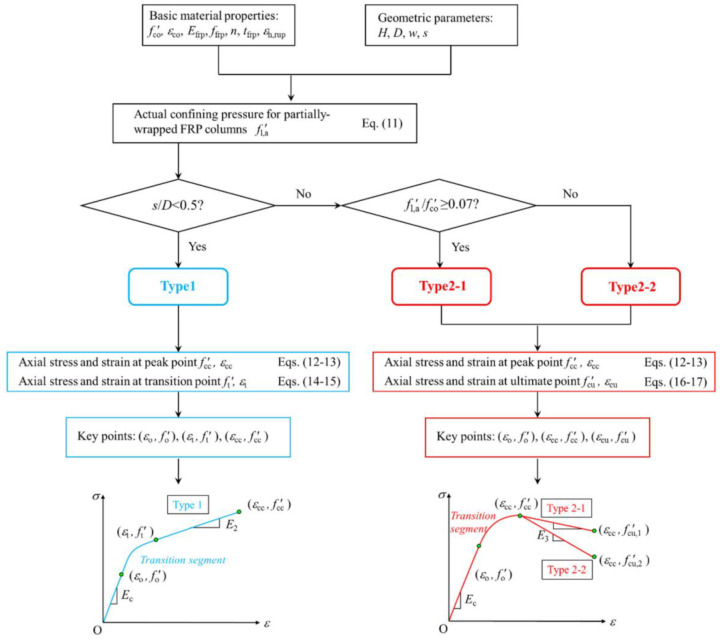
Modeling by Yang et al. (Equations (12)–(17) see literature [102]).

This model is illustrated in Figure 6:

Shayanfar et al. [103,104,105,106] developed new models to predict the expansion behavior of fully and partially FRP-constrained perimeter-compacted concrete columns under axial compressive loading. They proposed a new relationship between Poisson’s ratio and axial strain, taking into account the vertical arching phenomenon and the distribution of concrete expansion along the column height, which affects perimeter stresses. Additionally, they introduced a constrained stiffness index based on the constrained efficiency factor.

**Figure 7:**
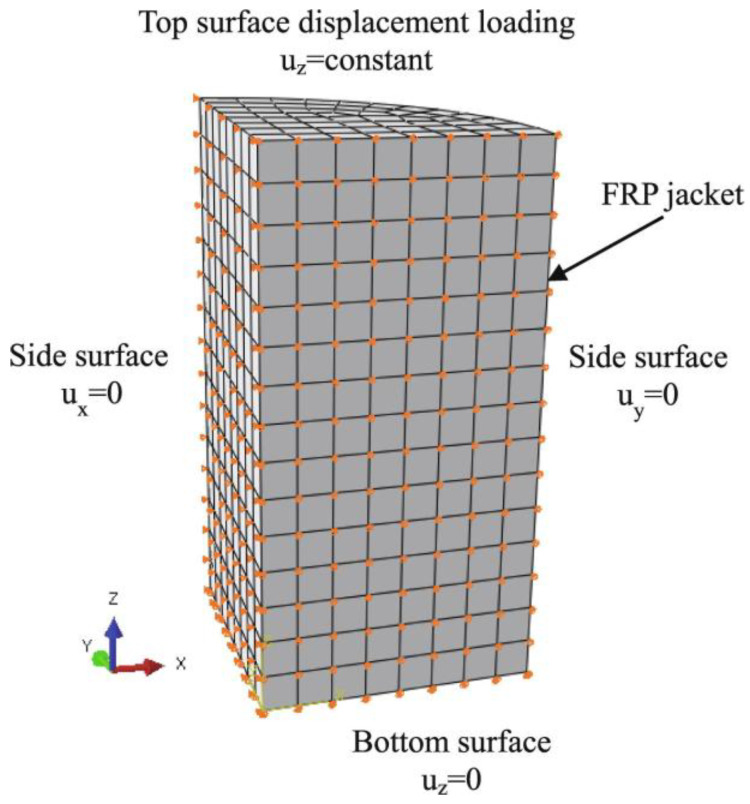
Finite element model of FRP-confined circular column [107].

**Figure 8:**
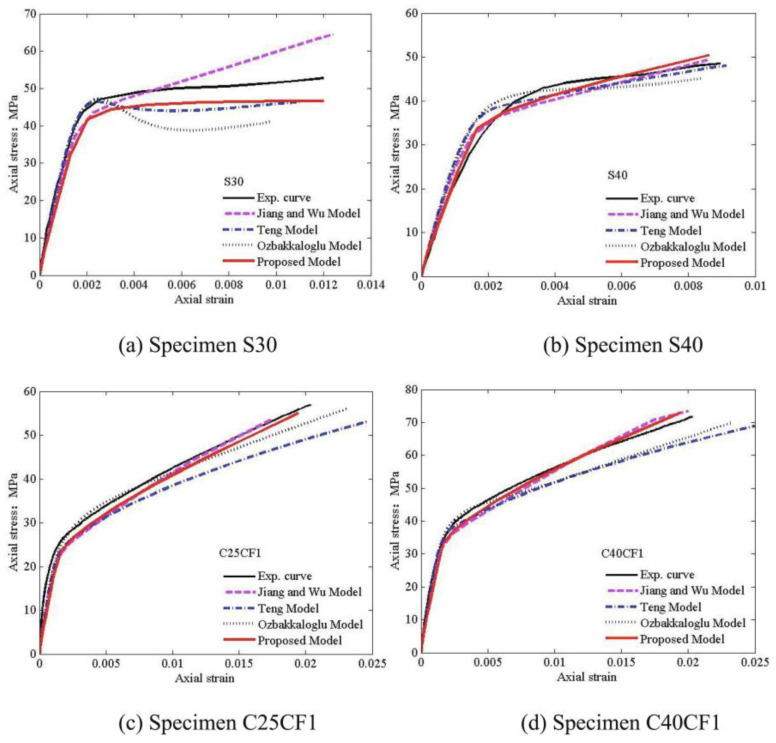
Comparison of FE and experimental results in FRP-confined circular columns [107] (Wu [108], Teng [109], Ozbakkaloglu [110]).

Li et al. [107] established an intrinsic model for non-uniform passively restrained concrete based on the true triaxial testing of passively restrained concrete cubes. They determined the main parameters of the plastic damage model, such as damage variables, hardening/softening rules (cohesive stresses), and flow rules (expansion angle). As can be seen from Figure 7 and Figure 8, the model, validated through ABAQUS simulations, has been successfully applied to analyze FRP-constrained cylindrical and square columns, both fully and partially restrained.

Wang et al. [111] investigated the compression behavior of partially FRP-constrained concrete and proposed an ultimate strain model that accounts for strain localization. According to their model, the global ultimate strain ε_cc_ can be determined from the cross-sectional area of effectively constrained concrete, the stress–strain path of the wrapped concrete, and the ultimate strength and strain of the wrapped concrete, as described by Equation (54).

(54) $$\varepsilon_{\mathrm{cc}}=\frac{\sum_{i=1}^{n}h_{i}\varepsilon_{c,i}}{H}+\frac{H_{1}\varepsilon_{c1}}{H}$$

H is the total height of the specimen, H1 is the total height of the wrapped area and is the ultimate strain of the wrapped concrete, and hi is the depth of each concrete strip and is the axial strain of the concrete strip i.

Wang et al. [112] developed a new analysis-oriented model for partially FRP-wrapped RC columns. Their model improves upon existing well-known expansion and active confinement models by considering the effects of the FRP wrapping scheme and the interactions between the FRP strips and steel hoops/helixes.

**Figure 9:**
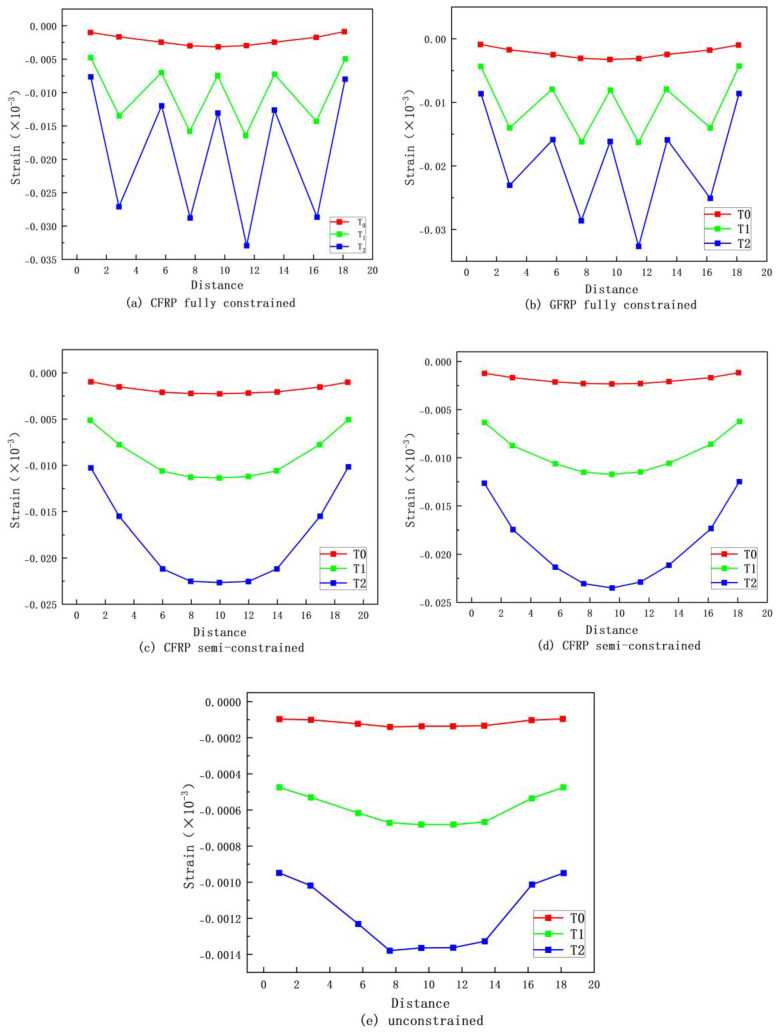
The longitudinal strain corresponding to the different time points and positions [113] (T0 denotes before compression, T1 denotes during compression, and T2 denotes when compressed to failure).

Yongcheng Ji and Yunfei Zou [113] conducted tests on partially restrained concrete with FRP fibers, employing finite element analysis (FEA) and digital image correlation (DIC) techniques. They observed distinct differences in the longitudinal strain distribution between the surfaces of fully and partially restrained specimens. Specifically, fully restrained specimens exhibited a parabolic longitudinal strain distribution along the height direction, while partially restrained specimens showed an “M”-shaped distribution. The height strain images from their study are shown in Figure 9.

The databases used in these studies and the validation of their models are summarized in Table 3. (Note: all references to “fully” and “partially” in the table refer to fully confined and partially confined concrete, respectively. Analytical methods include regression analysis (RA), constitutive modeling (CM), and finite element analysis (FEA). The validation set is excluded from the dataset in Table 3 and Table 4, and the dataset in Table 5 includes all the data.)

Where the dataset is the data used for regression modeling or modeling and the validation set is the data used for validating the model.

**Figure 10:**
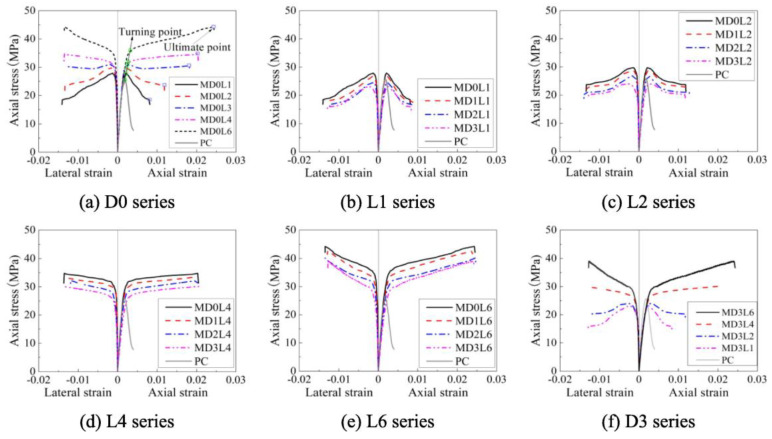
Stress-strain curves of BFRP-repaired concrete under monotonic compressive loading (symbols D0, D1, D2, and D3 denote 0%, +100%, −90%, and −80% of axial compressive stress, and L1, L2, L3, and L4 are for one-, two-, three-, and four-layer FRPs) [94].

For concrete under eccentric loading, pre-damaged concrete, and repairing pre-damaged concrete, Ma et al. [114] found that pre-damage adversely affected the strength and initial axial stiffness of repaired columns. However, the ultimate axial strain was minimally affected by the pre-damage. They concluded that the FRP cladding did not significantly contribute to the improvement in the initial axial stiffness of damaged RC columns, and other repair/reinforcement methods—such as epoxy injection, steel cages, and cross-sectional enlargement by adding new concrete—should be employed to improve the axial stiffness of pre-damaged columns. Moreover, they built upon the strength model, ultimate strain model, and stress–strain relationship models proposed by Mander [115] and Wang [116] to predict the compressive response of BFRP-repaired RC columns. They found that, except for the smallest specimen affected by wall effects, the ultimate strength of BFRP-constrained concrete decreased with an increasing size and slenderness ratio. Additionally, the perimeter pressure decreased with an increasing size and slenderness ratio of the BFRP, but increased with the severity of concrete pre-damage. Taking the size, slenderness ratio, and pre-damage effects into account, they developed models for uniaxial and cyclic axial compression of BFRP-restrained concrete [93]. They also estimated strength and strain at the limit state, accounting for the adverse effects of pre-damage levels, and proposed a monotonic stress–strain relationship for BFRP-repaired concrete prisms, as shown in Figure 10 [94].

Cao et al. [101] proposed an analytically-oriented stress–strain model and a transverse strain-axial strain model for FRP-constrained pre-damaged concrete columns, incorporating the factor of the fillet radius ratio. A new model for the stress–strain relationship of FRP-constrained pre-damaged concrete under eccentric loading was developed by Tijani et al. [117]. Their study considered a wide range of eccentricities, covering specimens with an eccentricity-to-radius ratio (e/R) from 0 to 0.67, a range that is also applicable to most existing studies on eccentrically loaded FRP-restrained concrete columns. For the pre-damage parameter, the damage levels ranged from 0 to 0.23, with damage levels ranging from 0% to 100%, including a damage level of −75% that approximated complete failure. This work examined the full range of concrete pre-damage levels. Subsequently, they successfully extended this stress–strain model to non-uniformly confined concrete cubes [118]. Zheng et al. [119] developed a plastic intrinsic model for concrete under general 3D compressive stresses, incorporating evolved eccentricity potential surfaces. This model accurately captured existing compression test results of concrete cubes under non-uniform passive constraints.

Wang et al. [120] experimentally found that a full wrap strategy is more effective than CFRP strips for columns under small eccentricity. However, increasing the number of longitudinal CFRP layers on the tensile side of the specimen can lead to better reinforcement outcomes for specimens subjected to large eccentricity. They also proposed a new stress–strain model for FRP-constrained concrete considering the preload effect.

Lu et al. [121] proposed a model to evaluate the expansion behavior and axial stress–strain response of FRP-constrained concrete under preload by considering the preload force. Modeling a series of rectangular CFRP-constrained RC columns with different cross-sectional dimensions, Fan et al. [122] observed that the damage mode changed from brittle compression damage to ductile tensile damage as the load eccentricity increased. This transition occurred consistently across rectangular columns with different cross sections, although the crack width became significantly larger as the cross-sectional size increased. Regarding the relationship between axial load and medium-to-high deflection, the fillet radius significantly influenced the maximum axial load and the softening phase, especially at smaller load eccentricities. Considering the influence of dimensional effects, the design equations proposed by ACI 440.2R-17 were modified by Fan et al. Wang et al. [100] developed a design-oriented axial stress–strain model for prestressed CFRP-reinforced concrete columns based on basic curve analysis assumptions.

The studies on eccentric loading and prestress damage are summarized in Table 4.

**Table 4:** Data table for eccentric loading and prestress damage studies.

Researchers	Research Method	Fiber Type	Obligatory Object	Cons Type	Variate	Data Set/ Validation Set	Statistical Indicators	
Ma et al. [114]	RA	BFRP	RC Cylinder	fully	Pl, n, Cl	22/	/	
Ma et al. [93]	RA	BFRP	Cylinder	fully	Sr, size	70/75	R^2^	
							Strength	0.92
							α_d_	0.91
Ma et al. [94]	RA	BFRP	Prism	fully	n, Lot, Pl	62/8	R^2^	
							Turning point compressive stress	0.80
							Turning point compressive strain	0.65
							Limiting point compressive stress	0.87
							Limiting point compressive strain	0.76
Cao et al. [101]	CM	/	Cylinder, Square column	fully	Shape, Pl, Cs	313/	/	
Tijani et al. [117]	CM	CFRP	Cylinder	fully	e_0_, Pl	72/	/	
Zheng et al. [119]	FEA	/	Square column	fully	shape	/16	/	
Wang et al. [120]	RA	CFRP	RC Square column	fully and partially	e_0_, Pl, pos	12/67	R^2^	
							Strength	0.926
							Strain	0.961
Fan et al. [122]	FEA	CFRP	RC Square column	fully	e_0_, Ra	/18	/	
Wang et al. [100]	CM	CFRP	Cylinder	fully	n, Pll	/37	R^2^	
							Strength	0.931
							Strain	0.932

(1) α_d_: strength reduction factor; (2) Pl: pre-damage level; (3) Sr: length-to-slenderness ratio; (4) Lot: load type; (5) Cs: FRP constraint stiffness; (6) e_0_: eccentricity; (7) Ra: radius of rounded corners; (8) Pll: prestressing level.

It is evident that repairing pre-damaged concrete is crucial. The main techniques for repairing FRP-confined pre-damaged concrete columns include fiber-reinforced cementitious matrix (FRCM) overlays [123,124], post-tensioned prestressed FRP strengthening [125], local multi-layer FRP wrapping for the damaged areas [126,127], sandblasting treatment or the use of interface agents (e.g., epoxy primer) to enhance the bond between FRP and damaged concrete [128,129], combining FRP with thin steel plates (fixed by bolts or adhesives) in the damaged region to suppress crack propagation using the high stiffness of the steel plates [130,131,132], and synergistically improving the load-carrying capacity by introducing shape memory alloys (SMAs) or self-healing concrete to dynamically adjust the confinement stress or automatically repair microcracks [133,134]. These methods not only enhance the structural load-bearing capacity but also improve its durability and long-term performance, offering valuable technological solutions for structural repair and strengthening.

**Figure 11:**
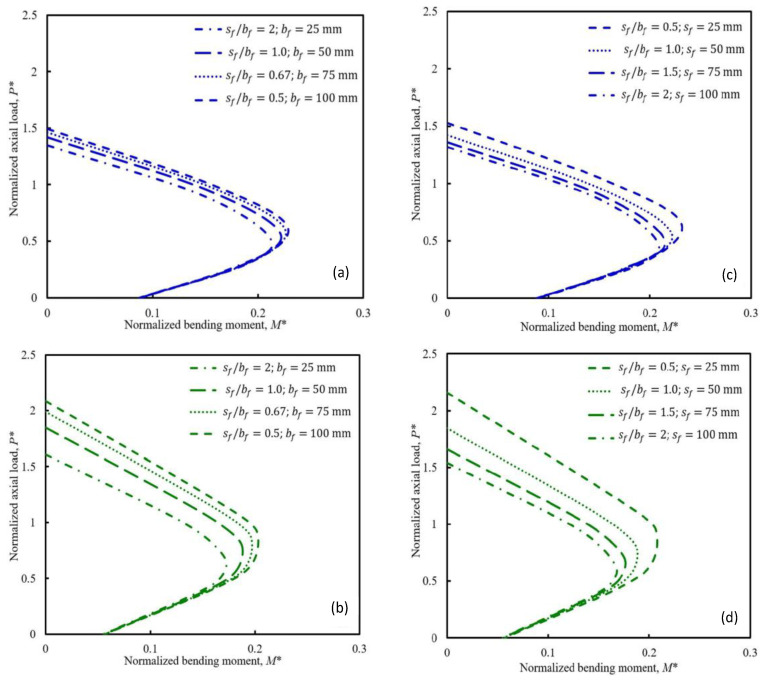
Effects of gap (sf) on the envelope of (**a**) discrete CFRP-wrapped square columns and (**b**) discrete CFRP-wrapped columns; CFRP bandwidth (bf) on the envelope of (**c**) discrete CFRP-wrapped square columns and (**d**) discrete CFRP-wrapped columns [94]. (The normalized axial load (P*), the corresponding normalized bending moment (M*)).

In addition to the aforementioned influencing factors, the size effects, strain rate, and spacing and confinement ratio of FRP are also significant determinants of the behavior of FRP-constrained concrete systems. Mai et al. [97] conducted parametric investigations to assess the effect of different parameters on the damage envelopes of square and circular RC columns with discrete CFRP constraints. As Figure 11 shows, they found that the clearance spacing, bandwidth, and CFRP confinement ratio of the CFRP bands significantly affected the damage envelopes of square and circular RC columns with discrete FRP confinements. At a fixed gap spacing and bandwidth ratio, changes in the CFRP bandwidth had a negligible effect on the damage envelopes of discrete CFRP-constrained square columns but had a slight impact on the damage envelopes of discrete CFRP-constrained circular columns. However, under the same amount of CFRP, discrete FRP-constrained concrete columns with a smaller net spacing of CFRP bands exhibited higher damage envelopes.

**Figure 12:**
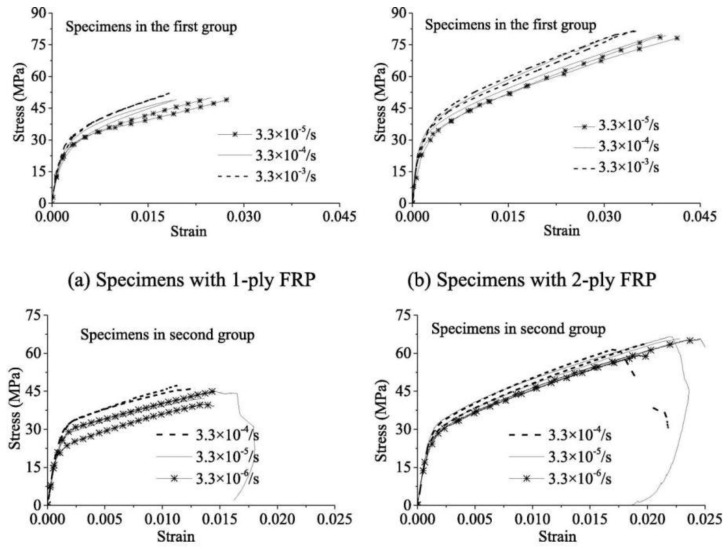
Stress–strain curves with different strain rates (0.6-ply,1-ply, and 2-ply means 0.6-layer, 1-layer, and 2-layer FRP) [136].

Hao et al. [135] proposed a passive stress–strain model based on fundamental interacting bond-slip and shear-friction mechanisms, incorporating size effects. Cao et al. [136] investigated the mechanical behavior of FRP-constrained concrete cylinders under uniaxial compression at varying strain rates, based on earlier studies [137,138,139] and proposed a model that can be used to predict FRP-constrained circular concrete columns under strains ranging from 3.3 × 10^−6^ to 3.3 × 10^−3^/s. The stress–strain curves at different strain rates are shown in Figure 12.

To examine the strength of FRP-constrained damaged and undamaged concrete columns, Zhang et al. [140] collected 900 test data points from the published literature and proposed a model for undamaged, load-damaged, and fire-damaged circular and square columns using the Hoek–Brown failure criterion. Chen et al. [141] found that the stress and strain capacity of restrained concrete increased with the increasing FRP restraint. However, they also observed that increasing the cross-sectional aspect ratio and using high-strength concrete negatively affected the axial behavior of the inner concrete due to an increasingly inhomogeneous FRP circumferential pressure distribution. They also compared the effects of elliptical-fiber-reinforced polymers (FRPs) to circular FRPs, introduced a shape factor, and developed a design-oriented elliptical FRP-constrained concrete model. This model investigated the behavioral characteristics of FRP-constrained elliptical concrete under cyclic compression loading [142].

Saleh et al. [143] aimed to address the poor effectiveness of FRP wrapping in improving the strength and ductility of slender columns and the increased likelihood of column buckling post-wrapping. They derived a predictive model to define the limits of the slenderness ratio for FRP-confined rectangular columns, using an accurate design-oriented stress–strain relationship for FRP-confined rectangular cross-sections, alongside an analytically derived loss of column capacity relationship.

Liao et al. [144] found that the desired confinement effect in a specimen could be achieved by varying key confinement parameters, including the helix angle, width, and the number of FRP strip layers. Their study demonstrated that increasing the width and thickness of the FRP strips enhanced the ultimate axial stresses and strains of FRP-helical-stripped confined concrete, with a decrease in the helix angle having a more pronounced effect on the enhancement of ultimate axial stresses than on ultimate axial strains. Based on these findings, they proposed a new design-oriented stress–strain model for FRP-helical strip restrained concrete.

**Figure 13:**
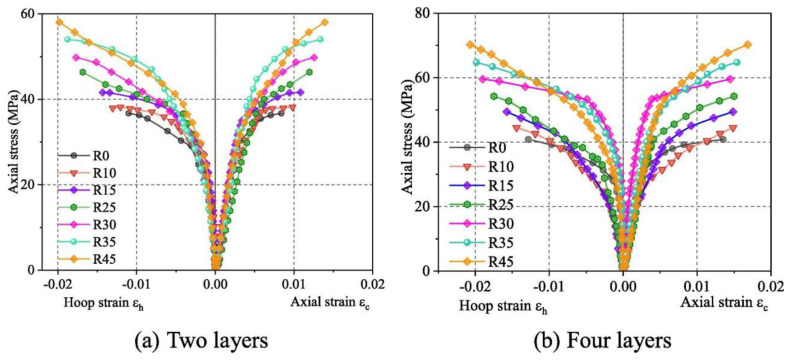
Stress–strain response of different BFRP layers (R is the radius of the fillet) [95].

Nadia Diboune et al. [98] incorporated parameters, such as the aspect ratio and shape factor, proposing a new stress–strain relationship that considers constraint parameters, such as the wrapping angle and depth of rectangular cross-sections. This model predicts the ultimate strength, strain, and axial stress–strain characteristics of CFRP-reinforced square and rectangular concrete columns. Xie et al. [95] discovered that the stress–strain curves of BFRP-restrained reinforced concrete square columns were bilinear, with the primary differences in restraint levels observed in the inelastic range. They also noted that the uniformity of circumferential pressure improved with an increase in the fillet radius. As a result, they proposed a prediction equation for the strain efficiency factor to improve the accuracy of existing strength models for square sections. The stress–strain curves for different BFRP layers are shown in Figure 13.

Gharaei et al. [145] proposed modification factors that can be applied to existing prediction models in the presence of a tilted fiber orientation to improve the prediction accuracy of the stress–strain relationship for FRP-constrained concrete cylinders. In their study, two prediction models were modified. The coefficient of determination R^2^ of the stress model was changed from 0.797 to 0.962 via modification of Arabshahi et al.’s model [146], while the coefficient of determination for the strain model R^2^ increased from 0.509 to 0.744, and the model of ACI 440.2R [147] was modified to change the coefficient of determination R^2^ of the stress model from 0.777 to 0.934 and the coefficient of determination R^2^ of the strain model from 0.227 to 0.091.

Li et al. [148] investigated the effect of dimensions on circular concrete columns reinforced through BFRP reinforcement and helical reinforcement. They developed a size-dependent stress–strain model that accounts for dimensional effects, such as the restraint strength, corresponding strain, and deterioration rate, which are essential for evaluating the restraint behavior of FRP-reinforced concrete columns.

To explore the dynamic compression behavior of FRP-constrained concrete, Jiang et al. [149] proposed an analytical model for dynamically loaded FRP-constrained concrete columns by incorporating the strain rate effect of concrete, the inertial confinement effect, and the strain rate effect of FRP into the static compression stress–strain model.

Chen et al. [150] aimed to improve the strain efficiency of FRP sheaths by proposing an FRP spiral strip wrapping scheme. Uniaxial compression tests were carried out on four cylindrical specimens of varying sizes, and the PIV (Particle Image Velocimetry) technique was used to capture circumferential and axial strains, as well as the strain distribution. The test results demonstrated that the FRP ring fracture strains, measured using PIV, were in close agreement with those observed in the specimen tests. Chen et al. subsequently modified the existing FRP-constrained concrete stress–strain model to better predict the test results.

**Figure 14:**
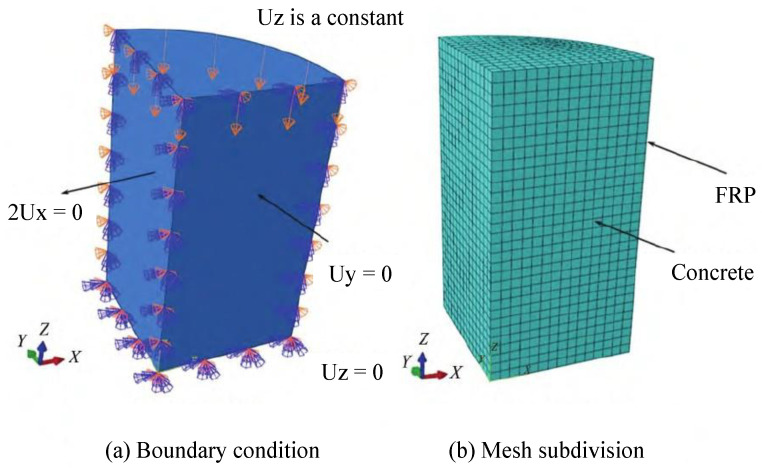
Finite element simulation [151].

**Figure 15:**
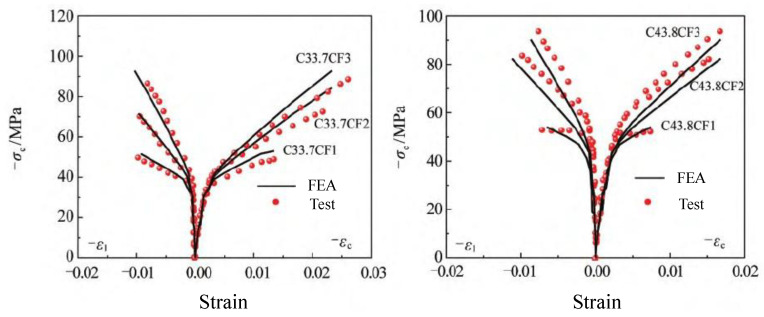
Comparison of FEA and test stress–strain curves [151] (Xiao et al. [31]).

Zhang et al. [151] proposed a modified model based on the conventional Drucker–Prager model, incorporating the yield criteria, hardening and softening conditions, and flow laws necessary for analytical modeling. This model was specifically developed to predict the mechanical behavior of FRP-constrained circular-section concrete columns under uniaxial loading. As shown in Figure 14 and Figure 15, once the constrained stiffness ratio of a given FRP-constrained concrete specimen is determined, the stress–strain curve can be derived through finite element analysis.

Zhou et al. [152] explored the cyclic compression behavior of FRP-reinforced concrete columns, examining the effects of parameters, such as the restraining stiffness (e.g., helical pitch), reinforcement ratio, and loading scheme. Their experimental study showed that FRP longitudinal reinforcement could significantly reduce the plastic strain of concrete in FRP-reinforced concrete columns. They also found that the axial load–strain curves exhibited a three-phase behavior, with the FRP longitudinal bars playing a crucial role in resisting axial stresses. Based on their findings, Zhou et al. proposed a new FRP-RC model to predict the axial cyclic behavior of FRP-reinforced concrete columns.

Wu et al. [153] developed a computational model coupled with the FDM (Finite Difference Method) and DEM (Discrete Element Method) to simulate the axial compression test of FRP-constrained concrete specimens. They found that the ratio of the linear modulus to the bond modulus affects the post-peak strength of FRP-constrained concrete. Additionally, the linear modulus between the concrete and the sheath influences strain distribution in the sheath. They noted that adjusting the internal friction angle is necessary to make the modeling more realistic.

Sun et al. [154] concluded that accurately predicting the strength and ductility of FRP-reinforced rectangular RC columns requires a precise evaluation of the interaction between FRP constraints and the buckling behavior of reinforcement at any loading level. They proposed a combined beam model and a tension-bending beam model to evaluate the lateral support stiffness of reinforcement, considering both the FRP-encapsulated concrete cover in the corner region of rectangular columns and in-plane reinforcement.

**Figure 16:**
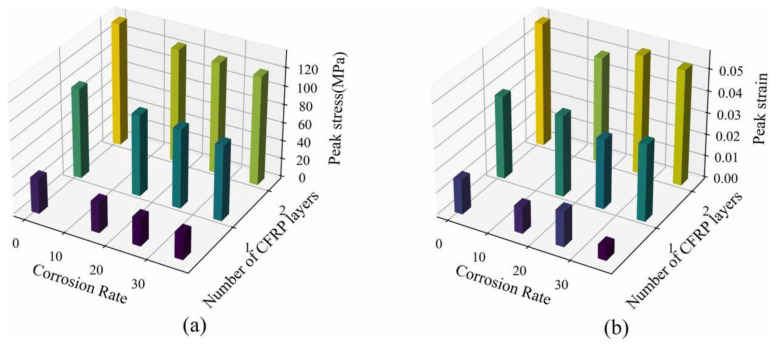
Variation in peak load carrying capacity (peak stress, peak strain) with the corrosion rate and number of layers of the CFRP [99]. (**a**) peak stress (**b**) peak strain.

Li et al. [99] explored the effects of the number of CFRP layers and corrosion rate on stress and strain, considering the confinement mechanism of the hoop and CFRP and the degradation of CFRP fracture strain (Figure 16). They proposed an experimental and predictive model for the axial-pressure response of CFRP-reinforced corroded RC cylinders.

**Figure 17:**
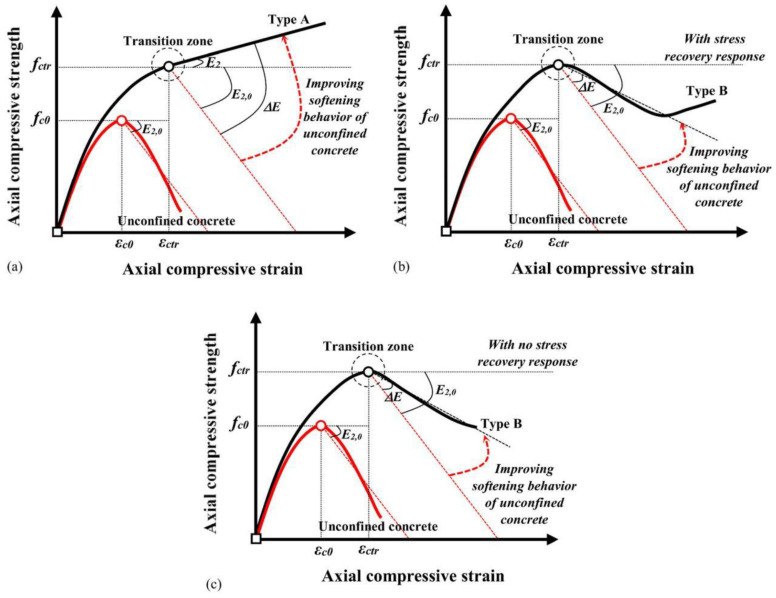
Typical stress–strain behavior; (**a**) fully strain-hardened behavior (Type A); (**b**) strain-softened-hardened behavior (Type B); (**c**) fully strain-softened behavior (Type B) [156].

Zhong et al. [155] proposed a stress-path-dependent model for FRP-constrained concrete based on the energy balance method, which accounts for the stress path by considering the additional work performed by the active constraint pressure in the stress-path-independent model. Shayanfar et al. [156] developed a new design-oriented stress–strain model to determine the softening–hardening stress–strain behavior of FRP-constrained concrete columns of the general cross-section, which is unified for both circular and non-circular columns. For modeling, they introduced a new index based on the dimensionless constrained stiffness, below which the column response transitions from fully hardened behavior (Type A) to post-peak strain-softened-hardening behavior (Type B), as shown in Figure 17. A parabolic–linear stress–strain formulation was proposed for Type A columns, with a new expression for calculating the slope of the linear hardened branch. For Type B columns, a novel method was introduced to model the stress relief–recovery response outside the transition zone.

A summary of the research on other influencing factors is shown in Table 5:

**Table 5:** Summary of compressive stress studies.

Researchers	Research Method	Fiber Type	Obligatory Object	Cons Type	Variate	Data Set	Statistical Indicators	
Mai et al. [97]	CM	CFRP	Square and round RC columns	partially	St, bf, Cr	16	/	
Hao et al. [135]	CM	/	Rectangular and circular RC columns	fully	size	23	/	
Cao et al. [136]	CM	CFRP	Cylinder	fully and partially	pos, Str	28	IAE = 0.175	
Zhang et al. [140]	CM	/	Cylinder, Square column	fully	shape, pt, pl, Cs	900	IAE = 0.106	
Chen et al. [141]	CM	/	Elliptic cylinder	fully	Cq, csar, fc’	73	R^2^	
							Strength	0.920
							Strain	0.816
Chen et al. [142]	CM	/	Elliptic cylinder	fully	csar, n, Lot	25	/	
Saleh et al. [143]	CM	/	Rectangular column	fully	Sr	200	λ_10%_	0.92
							λ_5%_	0.93
Liao et al. [144]	CM	/	RC Cylinder	fully	ha, bf, n	40	AAE	
							Strength	0.07
							Strain	0.13
Nadia Diboune et al. [98]	RA	CFRP	Square, Rectangular columns	fully	csar, shape, h	360	R^2^	
							Strength	0.81
							Strain	0.81
Xie et al. [95]	CM	BFRP	RC Square column	fully	Ra, n	272	AAE = 0.07	
Li et al. [148]	RA	BFRP	RC Cylinder	fully	d, sndb	36	Cov	
							Strength	0.07
							Strain	0.22
Jiang et al. [149]	CM	/	Cylinder	fully	n, Str	45	R^2^	
							Limiting axial stress	0.78
Chen et al. [150]	CM	CFRP	Cylinder	fully	d, size	24	/	
Zhang et al. [151]	CM, FEA	/	Cylinder	fully	n, fco’	57	R^2^	
							Strength	0.93
							Strain	0.92
Zhou et al. [152]	CM	/	RC Cylinder	partially	Rr, St, Lot	24	Unloading strain	R^2^ = 0.99
							Plastic strain	R^2^ = 0.97
Li et al. [99]	CM, RA	CFRP	RC Cylinder	fully	Cde, n	80	MRE	
							Strength	14%
							Strain	32.6%
Zhong et al. [155]	CM	/	Cylinder	fully	Ucc, d, t	369	AAE	
							Strength	0.087
							Strain	0.311

(1) λ_10%_: a 10% reduction in capacity; (2) λ_5%_: a 5% reduction in capacity; (3) COV: coefficient of variation; (4) MRE: average relative error; (5) Cr: constraint ratio; (6) Str: strain rate; (7) Pt: injury type; (8) Cq: FRP constraints; (9) csar: cross-section aspect ratio; (10) ha: spiral angle of FRP strips; (11) Rr: rebar rate; (12) Cde: corrosion degree.

#### 3.2.2. Machine Learning to Predict Compressive Performance of FRP-Constrained Concrete Columns

In recent years, several researchers have employed neural networks to predict the compressive strength, with common machine learning prediction methods, such as ANN, extreme gradient boosting (XGBoost), random forest (RF), and GEP. Naderpour et al. [157] and Raza et al. [96] have predicted the effect of the confinement of FRP concrete columns by ANN. Milad et al. [158] introduced three versions of an integrated machine learning model—XGBoost, multivariate adaptive regression spline (MARS), and RF—for strain prediction of FRP-restrained concrete. The results revealed that the MARS model provided superior prediction accuracy. However, the RF model demonstrated better performance when using strain as the sole input parameter for predicting the FRP composite strain enhancement ratio.

Ilyas et al. [159] proposed an updated formulation to predict the confined compressive strength (fcc), considering the most influential parameters. The developed GEP model was evaluated using various statistical and investigative methods, effectively capturing results that were more accurate and closer to the actual response by incorporating a large number of input variables. Fallah Pour et al. [160] predicted the limiting conditions and transition points of the axial stress–strain curve for FRP-cloth-restrained concrete using a meta-heuristic algorithm. They compared the effectiveness of this method with the GP approach and found that the GP method yielded more accurate results in determining these critical parameters.

Chen et al. [161] considered 298 experimental data points of FRP-restrained circular concrete columns and used six parameters, such as the diameter-to-fiber thickness ratio (D/t) and tensile strength of FRP (ffrp), as input sets. They compared existing models with experimental data and found that ANN and support vector regression (SVR) could effectively predict the compressive strength and peak compressive strain of FRP-constrained concrete columns, offering a reliable method for predicting the mechanical behavior of such columns.

Hanteh et al. [162] employed an AI-based computational model to estimate the mechanical behavior of FRP-wrapped circular concrete columns. Using adaptive multiple regression and extreme machine learning methods for modeling, the results showed that the combined MARS-PSO model provided superior performance, with correlation coefficients of 0.9972 during both the training and testing phases.

Kumar et al. [163] explored the use of Gaussian Process Regression (GPR), support vector machine (SVM), ANN, Optimized SVM, and Optimized GPR models for predicting the compressive strength of FRP-constrained concrete cylinders. The results showed that the Optimized GPR model outperformed all other models in terms of prediction accuracy.

Cakiroglu et al. [164] trained seven different machine learning models to predict the ultimate strain and compressive strength of AFRP-coated reinforced concrete. Among these models, the Extra Tree, XGBoost, and KNN models performed best in terms of predicting the ultimate strength and strain.

Peng et al. [165] developed a migration learning-based model to predict the confined strength of FRP-transversely reinforced concrete. The performance of the proposed model was compared with six machine learning models and seven physically based models, showing that the proposed model demonstrated high prediction accuracy.

Xu et al. [166] employed finite element modeling (FEM) in conjunction with machine learning methods to accurately predict the load-carrying capacity under concentric and eccentric loading conditions. The hybrid deep learning model proposed in their study achieved an average R^2^ value of 0.969.

Yue et al. [167] simulated elliptical concrete columns constrained by FRP sheaths using Finite Element Analysis (FEA) software (e.g., ABAQUS). They extended this analysis to estimate the compressive strength of elliptical FRP-confined columns, using four tree-based machine learning algorithms, including decision tree, random forest, gradient boosting, and XGBoost. The study demonstrated that XGBoost outperformed the other algorithms in terms of prediction accuracy, achieving an R^2^ of 0.95.

The specific parameters of the study are shown in Table 6.

**Figure 18:**
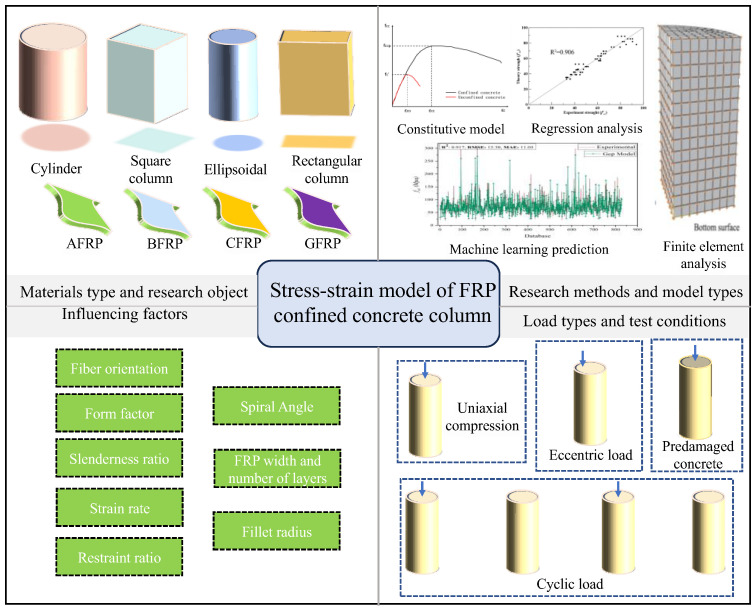
Model overview diagram [10,91,107,157].

In summary, the current research on the stress–strain model of FRP-constrained concrete is shown in Figure 18.

## 4. Discussions

This review aims to summarize recent studies on FRP-confined concrete columns under normal temperature conditions and, in conjunction with machine learning (ML) algorithms, provide insights and references for researchers in the fields of fiber-reinforced composites and materials science, while offering theoretical guidance for engineering practices.

1. Literature Selection: During the literature selection process, we prioritized studies based on their relevance to the research topic. We chose literature closely aligned with the subject matter and organized it according to the journal impact factor, ranking, and publication date to ensure that the cited references are of high quality and closely related to the topic. Additionally, we supplemented our search using specific keywords for targeted research areas, further expanding the breadth of the literature.

2. Content Overview: As a review paper, we focused on integrating and summarizing experimental designs, key findings, and model developments from various studies. We not only discussed the verification processes of existing models but also analyzed their potential improvements and adjustments. Furthermore, we proposed alternative solutions for similar problems, aiming to provide readers with a comprehensive understanding of the research progress in this field.

3. Data Visualization: In terms of graphical representation, we ensured the clarity of the figures and reconstructed some images. Data extracted from various scholars’ constitutive models were systematically listed in tables for easier comparison and reference.

4. Representative Study Selection: For studies with similar conclusions, we selected a few representative papers for a detailed analysis, while other relevant studies were referenced in the text. This approach highlights key research outcomes and provides readers with a broader background for a deeper understanding of the topic.

## 5. Conclusions and Outlook

### 5.1. Conclusions

This paper systematically reviews the progress in research on FRP-confined concrete columns, focusing on the interface bond performance, constitutive models under confinement, and predictive methods for compressive strength. It also explores the current application and potential of machine learning algorithms in the field. The main conclusions are as follows:

1. Performance and Application of FRP

FRP materials, with their excellent mechanical properties and durability, have become an important method for strengthening concrete structures in civil engineering. However, the mechanisms of FRPs under different confinement conditions require further in-depth research to refine the theoretical framework.

2. Current Status of Interface Bonding Performance Research

The bond performance between FRP and concrete interfaces is a key factor determining the effectiveness of strengthening. Despite a large number of experimental and theoretical studies, the existing bonding models lack uniformity and generality, and the quantity and quality of related experimental data need improvement.

3. Optimization and Limitations of Constitutive Models

Existing constitutive models for FRP-confined concrete are mostly based on empirical formulas and experimental data, effectively describing the axial compressive behavior. However, these models have limitations when describing performance under complex stress states, particularly in the characterization of nonlinear behaviors and failure mechanisms, which still offer room for improvement.

4. Application of Machine Learning in Research

Machine learning algorithms have shown great potential in predicting the mechanical properties of FRP-confined concrete. By appropriately selecting training datasets and input parameters, algorithms, such as ANN, SVR, and RF, have achieved high prediction accuracy. However, current studies are mainly limited to specific datasets, and the generalization ability and applicability of these models require further validation.

### 5.2. Outlook

Future research should focus on the following aspects:

1. Establishing more comprehensive and high-quality experimental databases to support the development and optimization of interface bonding performance and constitutive models for confinement.
2. Exploring multi-scale modeling methods that combine experiments, theory, and numerical simulations to enhance the physical interpretability of models.
3. A hybrid model combining traditional numerical analysis methods with machine learning techniques can improve the prediction accuracy and promote the integration of machine learning with traditional mechanics models. Specifically, numerical data generated from finite element analysis (FEA) can be used as input to train neural networks. The model’s performance is validated through cross-validation and test datasets to ensure its generalization ability and predictive accuracy. This approach aims to develop a more universal predictive framework, enhancing its applicability under complex stress conditions.
4. Developing standardized testing methods and evaluation systems to promote the internationalization and engineering application of FRP-related research.
5. Future research should focus on the performance of FRP-confined concrete under cyclic or dynamic loading conditions, which are critical for earthquake and impact-resistant design. Investigating how FRP materials behave under such loading conditions, including their stress–strain responses, bond performance, and failure mechanisms, will be crucial for improving the design of seismic infrastructure. Understanding the fatigue behavior of FRP-confined concrete, especially under cyclic loads, will enhance the resilience and durability of structures in earthquake-prone regions.

In conclusion, this review aims to provide theoretical references for further research on FRP-confined concrete and its practical engineering applications, while also guiding the expansion of machine learning methods in structural reinforcement fields.

## Figures and Tables

**Table 1:** Fundamental mechanical properties of FRP materials [5].

	CFRP	GFRP	AFRP	BFRP
Tensile Strength/MPa	1650–3000	517–1207	1200–2068	1500–2400
Modulus of Elasticity/GPa	152–165	41–55	50–74	90–130
Rate of elongation/%	1–1.5	3.5–5	2–2.6	1.2–1.6
Specific gravity	1.5–1.6	1.5–2.0	1.25–1.4	1.9–2.1

**Table 2:** Prediction of FRP-confining concrete bond strength using ML models.

Researchers	ML Model	Validation Set	Training Set	Test Set	Number of Input Layer Nodes/(Number)			Hidden Layers/Nodes	R
Mashrei [77]	BPNN	/	83%	17%	6	bc/(mm)	100–228	2/5	Train = 0.99
						fc’/(MP)	16–50		
						bf/(mm)	10–100		
						t/(mm)	0.08–1.4		Test = 0.99
						Efrp/(GPa)	83–300		
						Lb/(mm)	50–300		
Amin [90]	LIGHT GBM	/	70%	30%	5	Efrp*t/(Gpa*mm)	12.9–78.2	/	Train = 0.942
						bf/(mm)	30–60		
						fc’/(Mpa)	22.7–48.2		Test = 0.8
						Dg/(mm)	5–10		
						Wg/(mm)	5–15		
Wang [86]	RBFNN, LSVR	/	88%	12%	6	bc/(mm)	80–500	RBF neuron	(R^2^)
						fc’/(MP)	8–75.5		
						bf/(mm)	10–150		Train = 0.92
						t/(mm)	0.083–4		Test = 0.85
						Efrp/(GPa)	22.5–425		Test = 0.85
						Lb/(mm)	20–400		
Golafshani [78]	ANN, GP	15%	70%	15%	7	Pos	1–2	1/12	/
						Surf	1–3		
						db/(mm)	9.53–28.58		
						fc’/(Mpa)	23.43–48.86		
						C/db	1–6.2		
						l/db	3.56–97.24		
						Atr/sndb	0–0.08		
Köroğlu [80]	ANN	/	88%	12%	8	FRP type	1–3	2/30	R^2^ = 0.9
						Surf	1–3		
						Confining	1–2		
						db/(mm)	6.38–28.58		
						fc’/(Mpa)	4.29–8.08		
						C/db	0.92–9.34		
						l/db	2.5–115.79		
						Atr/sndb	0–0.15		
Zhang [84]	GEP, RF	/	69%	31%	5	Lb/(mm)	30–508	/	GEP
						EfAf/(kN)	1300–15,947		R_Train_^2^ = 0.871
						Dg/Wg	1–6.875		R_Test_^2^ = 0.800
						ffrp/(Mpa)	6–90.7		RF
									R_Train_^2^ = 0.962
						fc’/(Mpa)	19–64.8		R_Test_^2^ = 0.780

(1) Efrp: the elastic modulus of FRP; (2) t: the thickness of the FRP layer; (3) bc: width of concrete; (4) Lb: bond length; (5) surf: bar surface; (6) l: bar length; (7) db: bar diameter; (8) C/db: minimum cover to bar diameter ratio; (9) Dg/Wg: The groove depth-to-width ratio; (10) Atr/sndb: the ratio of the area of transverse reinforcement to the product of transverse reinforcement spacing, the number of developed bars and bar diameter; (11) ffrp: the tensile strength of FRP; (12) bc: width of concrete prism; (13) bf: width of FRP; (14) pos: cover the bar position; (15) EfAf: the FRP axial rigidity; (16) fc’: concrete compressive strength.

**Table 3:** Table of other research databases and models.

Researchers	Research Method	Fiber Type	Obligatory Object	Cons Type	Variate	Data Set/ Validation Set	Statistical Indicators	
Li et al. [91]	RA	AFRP	Cylinder, Square column	fully	n; w/c; shape	156/42	R^2^	
							1 layer	0.86
							2 layer	0.86
							3 layer	0.906
							4 layer	0.93
Huang et al. [92]	RA	BFRP	Cylinder, Square column	fully	Fty; n; shape	59/	R^2^	
							Cylindrical strength	0.98
							Square column strength	0.92
							Cylindrical strain	0.95
							Square column strain	0.90
Yang et al. [102]	RA	/	Cylinder	partially	St/d; f_l,a_′/Ucc	100/100	/	
Li et al. [107]	RA, CM	/	Cylinder, Square column	fully and partially	Ucc; Cl	117/26	Fully cylinder	IAE
							Axial direction	0.0499
							Partially cylinder	IAE
							Axial direction	0.0786
							Lateral direction	0.0661
							Square column	IAE
							Axial direction	0.0733
							Lateral direction	0.0926
Wang et al. [111]	CM	/	Cylinder	partially	bf; St; n	12/89	R^2^	
							Strain	0.906
							Strain localization	0.992
Wang et al. [112]	CM	CFRP	RC Cylinder	partially	Pos; n; bf; sndb	27/24	AAE	
							Strength	0.074
							Strain	0.137

(1) Cons type: constraint type; (2) n: number of layers of FRP; (3) St/d: distance between FRPs/concrete cylinder diameter; (4) fl,a′/Ucc: actual constraint ratio, actual maximum perimeter pressure of FRP partially constrained concrete specimen/unconstrained concrete strength; (5) bf: width of strips of FRP; (6) w/c: water-cement ratio; (7) Cl: constrained load; (8) IAE: integration of absolute value of error; (9) AAE: average absolute error.

**Table 6:** ML model prediction of compressive strength in FRP-constrained concrete columns.

Researchers	ML Model	VALIDATION SET	Training Set	Test Set	Number of Input Layer Nodes/(Number)			Hidden Layers/Nodes	Statistical Indicators	
Naderpour et al. [157]	ANN	20%	60%	17%	6	d/(mm)	/	1/11	MSE = 0.001	
						h/(mm)	/			
						t/(mm)	/			
						Ft/(Mpa)	/			
						Ucc/(Mpa)	/			
						Efrp/(Gpa)	/			
Raza et al. [96]	ANN	20%	60%	20%	5	d/(mm)	51–406	2/20	R^2^	
						h/(mm)	102–812		0.907	
						t/(mm)	0.09–5.9		RMSE	
						Efrp/(Gpa)	10–663		0.204	
						Ucc/(Mpa)	12.41–188.2			
Ilyas et al. [159]	GEP	/	70%	30%	5	d/(mm)	0~400	/	R^2^	
						h/(mm)	100~800		0.917	
						t/(mm)	0~6		RMSE	
						Efrp/(Gpa)	10~612		12.30	
						Ucc/(Mpa)	6.25~187.5			
chen et al. [161]	ANN, SVR	/	80%	20%	6	d/t	0–500	1/10	ANN	R^2^
						fco/(Mpa)	9.9–136.3		Strength	0.92
						εco/(uε)	1500–3000		Strain	0.87
						ffrp/(Mpa)	2000–4000		SVR	R^2^
						Efrp/(Gpa)	200–300		Strength	0.96
						εfrp/(uε)	3×10^−4^~4.5×10^−4^		Strain	0.94
Hanteh et al. [162]	MARS-PSO	/	70%	30%	6	d/(mm)	51–219	/	R	
						h/(mm)	102–438			
						Ucc/(MPa)	0.089–5.9		train	0.997
						t/(mm)	19.4–103			
						fl/(MPa)	2.33–94.57		test	0.996
						ffrp/(MPa)	229.76–3820.36			
Kumaret al [163]	OP-GPR	/	70%	30%	11	d/(mm)	50–310	/	R	
						fc’/(Mpa)	8–204			
						h/(mm)	100–1000			
						Ucc/(Mpa)	6.2–200		train	0.998
						Fty	1–4			
						n	0.3–14			
						bf/(mm)	15–1000			
						t/(mm)	0.05–3.9		test	0.992
						Of	1–2			
						Efrp/(Gpa)	21.3–251			
						ffrp/(MPa)	69–4580			
Peng et al. [165]	NTLA	/	80%	20%	7	Acore/(mm^2^)	19,855.7–360,000	/	R^2^	
						Ucc/(Mpa)	18.972–40.476			
						d/(mm)	6.4–20			
						St/(mm)	30–150		train	0.938
						ffrp/(MPa)	640–2551			
						Efrp/(Gpa)	44–157		test	0.909
						shape	1–2			

## Data Availability

No new data were created or analyzed in this study.
